# Chronic Systemic Curcumin Administration Antagonizes Murine Sarcopenia and Presarcopenia

**DOI:** 10.3390/ijms222111789

**Published:** 2021-10-30

**Authors:** Luisa Gorza, Elena Germinario, Lucia Tibaudo, Maurizio Vitadello, Chiara Tusa, Irene Guerra, Michela Bondì, Stefano Salmaso, Paolo Caliceti, Libero Vitiello, Daniela Danieli-Betto

**Affiliations:** 1 Department of Biomedical Sciences, University of Padova, 35131 Padova, Italy; elena.germinario@unipd.it (E.G.); maurizio.vitadello@bio.unipd.it (M.V.); chiara.tusa@studenti.unipd.it (C.T.); irene.guerra@studenti.unipd.it (I.G.); michela.bondi@unipd.it (M.B.); daniela.danieli@unipd.it (D.D.-B.); 2 Department of Biology, University of Padova, 35131 Padova, Italy; lucia.tibaudo@unipd.it (L.T.); libero.vitiello@unipd.it (L.V.); 3 Department of Pharmaceutical Sciences, University of Padova, 35131 Padova, Italy; stefano.salmaso@unipd.it (S.S.); paolo.caliceti@unipd.it (P.C.)

**Keywords:** senescence, aging, sarcopenia, Grp94, gp96, nNOS, dystrophin, melusin, satellite cells

## Abstract

Curcumin administration attenuates muscle disuse atrophy, but its effectiveness against aging-induced, selective loss of mass or force (presarcopenia or asthenia/dynopenia), or combined loss (sarcopenia), remains controversial. A new systemic curcumin treatment was developed and tested in 18-month-old C57BL6J and C57BL10ScSn male mice. The effects on survival, liver toxicity, loss of muscle mass and force, and satellite cell responsivity and commitment were evaluated after 6-month treatment. Although only 24-month-old C57BL10ScSn mice displayed age-related muscle impairment, curcumin significantly increased survival of both strains (+20–35%), without signs of liver toxicity. Treatment prevented sarcopenia in soleus and presarcopenia in EDL of C57BL10ScSn mice, whereas it did not affect healthy-aged muscles of C57BL6J. Curcumin-treated old C57BL10ScSn soleus preserved type-1 myofiber size and increased type-2A one, whereas EDL maintained adult values of total myofiber number and fiber-type composition. Mechanistically, curcumin only partially prevented the age-related changes in protein level and subcellular distribution of major costamere components and regulators. Conversely, it affected satellite cells, by maintaining adult levels of myofiber maturation in old regenerating soleus and increasing percentage of isolated, MyoD-positive satellite cells from old hindlimb muscles. Therefore, curcumin treatment successfully prevents presarcopenia and sarcopenia development by improving satellite cell commitment and recruitment.

## 1. Introduction

Curcumin, the bioactive polyphenolic extract of turmeric, acts differently among tissues on signal transduction and gene expression (pleiotropism) [1], and exerts beneficial or detrimental effects depending on dosage (hormesis) [2]. In the skeletal muscle, curcumin induces cytoprotection in myocyte precursors [3], stimulates injury-induced myofiber regeneration [4], and attenuates disuse atrophy [5,6]. Curcumin supplementation combined to physical activity appears to be safe and beneficial in humans [7].

Curcumin exerts relevant anti-aging effects by increasing mean lifespan [8]. Age-associated muscle wasting is a major problem in elderly people [9]. The decline in performance and fitness leads to increased risk of falls and progressive loss of functional independence in daily activities. The European Working Group on Sarcopenia in Older People defined sarcopenia by the presence of low appendicular lean mass/height^2^ and altered muscle strength, and presarcopenia by the presence of decreased muscle mass without impact on muscle force development and contraction [10]. Loss of muscle force in the absence of loss of muscle mass, defined as asthenia/dynopenia [11,12], also characterizes the aging muscle [11,13]. Increased oxidative stress [14,15] and decreased activity of muscle stem cells [16] are among the hypothetical mechanisms driving sarcopenia development and putative curcumin targets [4,5].

Actual effectiveness of oral curcumin administration against muscle atrophy and sarcopenia development remains questionable [17]. Positive results were obtained after systemic curcumin administration [5,17] or oral supplementation using formulations with improved bioavailability [6,18], associated or not with other countermeasures, such as exercise. In vitro and in vivo beneficial effects on muscle cells derived from the exposure to the least curcumin dosages that selectively increased the protein levels of the endoplasmic reticulum (ER) chaperone Grp94/gp96 [3,5]. In fact, curcumin-induced maintenance of the physiological levels of this chaperone in rat unloaded muscles is required to preserve localization of active nNOS molecules at sarcolemma, within the dystrophin-glycoprotein complex at costameres, and the consequent pro-trophic effect of NO [5,12].

Here we investigated whether curcumin counteracted sarcopenia development in male mice by administering it subcutaneously, as a formulation, at a 6-day-interval for 6 months, starting at age of 18 months. Two related C57BL mouse strains were treated in parallel, since sarcopenia shows strain, sex and muscle-related differences [19,20]. Treated and vehicle-treated mice were assessed for spontaneous mortality and for the presence of sarcopenic, presarcopenic and asthenic/dynopenic signatures in representative slow and fast hindlimb muscles, i.e., soleus and Extensor Digitorum Longus (EDL), respectively. Results not only showed relevant differences in strain- and muscle-specific response to aging, but also that curcumin exerted both general and muscle-specific beneficial effects.

## 2. Results

### 2.1. General and Muscle-Specific Effects of Chronic Curcumin Administration in Old C57BL Mice

Table 1 illustrates body weight (BW) and absolute and normalized muscle weight (MW) to BW of EDL and soleus of 6- and 24-month-old C57BL6J (6J) and C57BL10ScSn (10ScSn) male mice. Old muscles of the 6J strain did not show any sign of mass loss compared to 6-month-old adult ones, whereas those of the 10ScSn strain displayed a mild, significant atrophy (−15%, *p* = 0.04).

**Figure 1:**
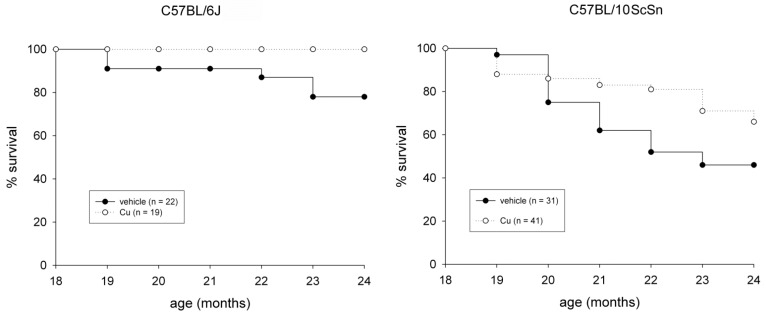
Survival plots of vehicle-treated (closed circle) and curcumin-treated (open circle) of C57BL6J and C57BL10ScSn mouse strains between 18- and 24-months of age. n indicates total number of mice for each group. Statistical analysis log-rank (Mantel-Cox) test *p* ≤ 0.04.

The subcutaneous administration of a curcumin formulation was performed every sixth day for 6 months, starting at 18 months of age on 60 mice from both strains. Fifty-three mice were treated in parallel with vehicle only. The procedure was well tolerated and no adverse effect was observed. Examination of liver cryosections from 24-old mice did not show treatment-dependent signs of toxicity, such as hepatocyte apoptosis and/or necrosis (not shown), nor chronic effects, such as the presence of a sustained endoplasmic reticulum stress-response or reactive hepatocyte proliferation, compared to those from vehicle-injected mice (Figure S1). Curcumin administration significantly reduced spontaneous mortality in both strains (Figure 1, *p* ≤ 0.04) and significantly counteracted loss of EDL and soleus muscle mass in 10ScSn mice (Table 1).

### 2.2. Effects of Aging and Curcumin Treatment on Muscle Contractile Properties

**Figure 2:**
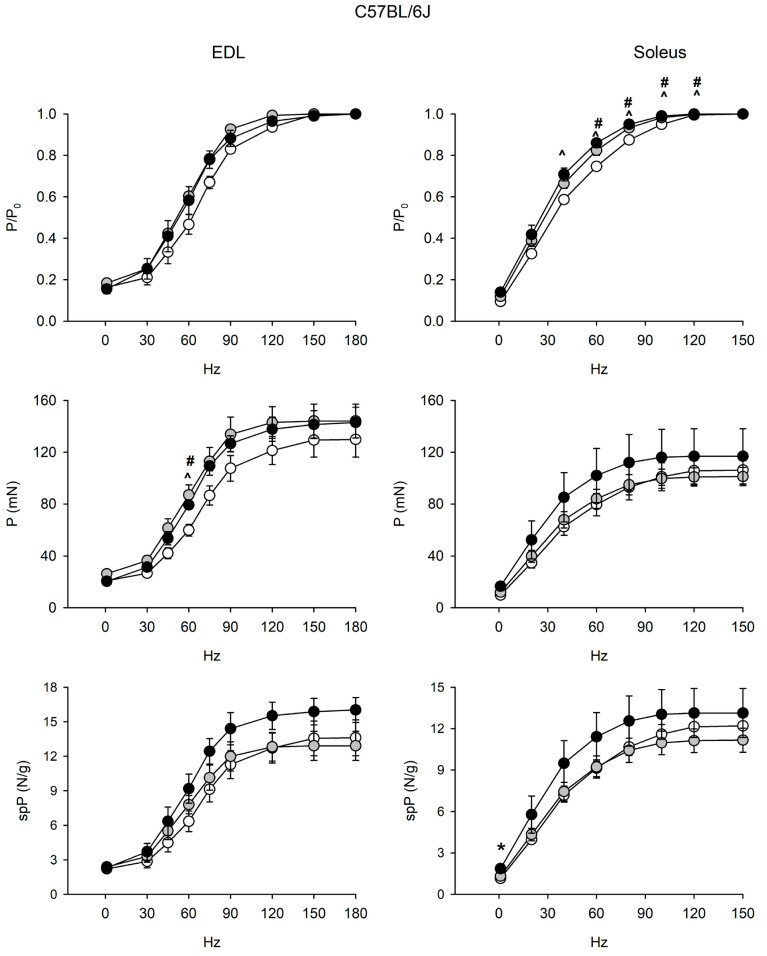
Force-frequency curves on adult (open circle), vehicle-treated old (gray circle) and curcumin-treated old (closed circle) EDL (left) and soleus (right) muscles of 6J mice. Muscles were stimulated at various frequencies under isometric conditions. Force is expressed as relative to the maximum tetanic force (top charts), as isometric absolute force (middle charts) and as specific tension (isometric absolute tension normalized to muscle wet weight) (bottom charts). Values are mean ± SEM. Significant difference (ANOVA and post-hoc *p* < 0.05) is indicated by * between old and curcumin-treated old, by # between adult and old, and by ^ between adult and curcumin-treated old. EDL data were obtained from n = 3 adult mice; n = 3 old mice and n = 4 curcumin-treated old ones; soleus data were obtained from n = 6 adult mice; n = 7 old mice, and n = 5 curcumin-treated old ones.

**Figure 3:**
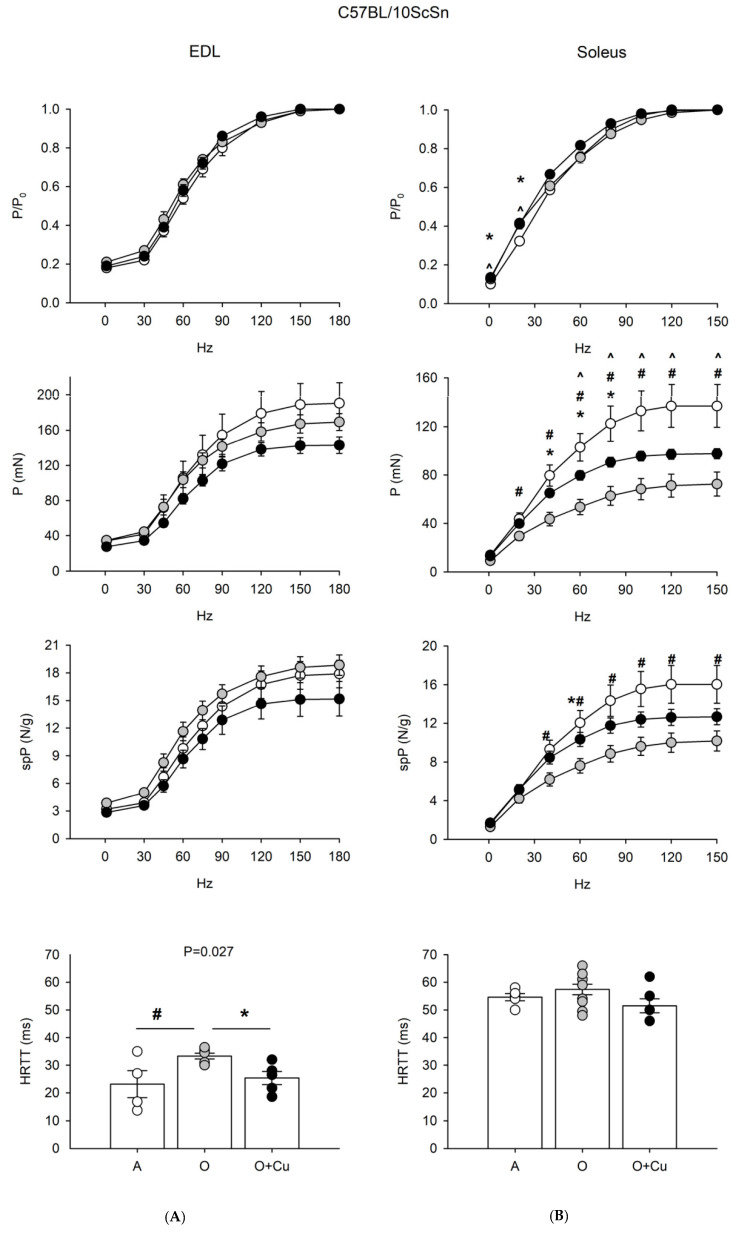
(**A**) Force-frequency curves on Adult (A, open circle), vehicle-treated Old (O, gray circle) and Curcumin-treated Old (O + Cu, closed circle) EDL (left) and soleus (right) muscles of 10ScSn mice. Muscles were stimulated at various frequencies under isometric conditions. Force is expressed as relative to the maximum tetanic force (top charts), as isometric absolute force (middle charts) and as specific tension (isometric absolute tension normalized to muscle wet weight) (bottom charts). Values are mean ± SEM. Significant difference (ANOVA *p* < 0.05) is indicated by * between O and O + Cu, by # between A and O and by ^ between A and O + Cu. (**B**) Histograms of half relaxation time of the tetanus (HRTT) of EDL and soleus muscles of 10ScSn mice. Values are mean ± SEM. Significant difference (ANOVA *p* < 0.03) is indicated by # between A and O *p* < 0.002) and by * between O and O + Cu (*p* < 0.007). EDL data were obtained from n = 4 adult mice; n = 7 old mice and n = 5 curcumin-treated old ones; soleus data were obtained from n = 5 adult mice; n = 10 old mice, and n = 6 curcumin-treated old ones.

Since muscle mass loss may be uncoupled from force loss during aging [9,10,11,12,13], senescence-related changes in contractility of EDL and soleus muscles were assessed in both strains (Figure 2 and Figure 3, Figures S2 and S3).

EDL and soleus muscles of 6J mice did not show age-related alterations of force and contractile properties (Figure 2 and Figure S2). Strikingly, relative force/frequency curves significantly shifted to the left in the old soleus.

EDL of 24-month-old 10ScSn mice showed only a significant increase of HRTT (*p* ≤ 0.03) that was blunted by curcumin treatment (Figure 3). Conversely, 10ScSn soleus muscle displayed aging-induced reduction of both absolute (20–150 Hz) and specific (40–150 Hz) tetanic force development (*p* < 0.05), in the absence of changes for other contractile parameters (Figure 3 and Figure S3).

Curcumin treatment significantly blunted the age-related decrease of specific tetanic tension (*p* < 0.05), and improved absolute tension development at frequencies between 40–80 Hz. However, the latter parameter still appeared significantly reduced at frequencies between 60–150 Hz, compared to adult mice (Figure 3).

### 2.3. Effects of Aging and Curcumin Treatment on Myofiber Number, Type and Size

**Figure 4:**
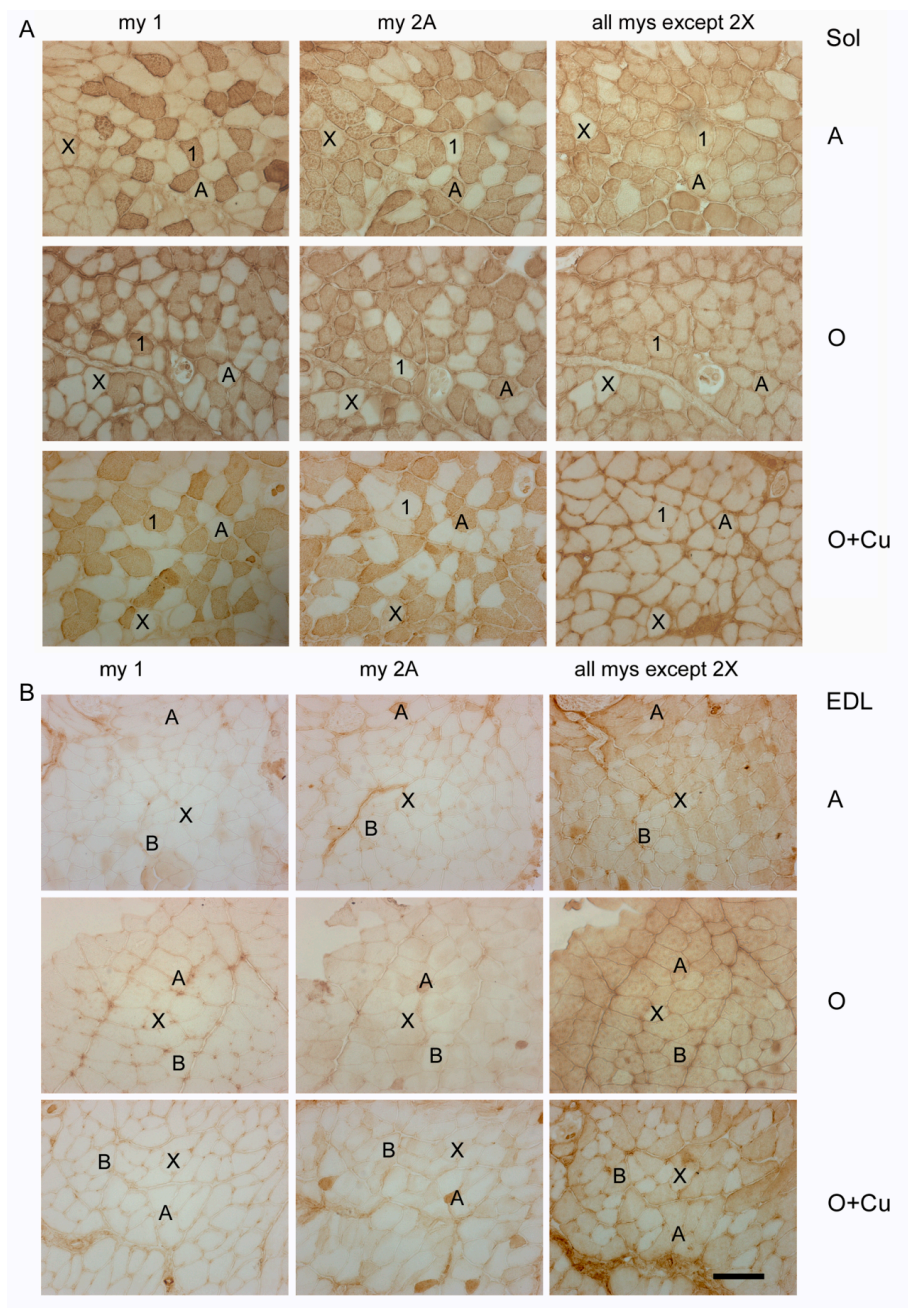
Representative immunoperoxidase stainings with anti-myosin (my) antibodies of serial cryosections of soleus (Sol) (panel (**A**)) and EDL (panel (**B**)) obtained from adult 10ScSn mice (A) and old ones, after either vehicle (O) or curcumin administration (O + Cu). Type-1 and -2A myofibers were identified after staining with anti-my-1 or anti-my-2A antibodies, respectively, whereas type-2X myofibers were identified indirectly, by the absence of staining with antibodies for my-1 and my-2A and with the BF-35 antibody, which reacts with every myosin type except 2X [21]. Type-2B myofibers were identified by the absence of staining for my-1 and my-2A and the presence of staining with BF-35 antibody. Bar: 100 μm.

Aging-induced strain- and muscle-specific effects on fiber-type composition, size and total number appeared variably blunted by curcumin treatment (Table 2 and Figure 4).

No major strain-related difference existed in fiber-type composition of adult soleus and EDL. In adult soleus, type-1 and -2A fibers predominate (Table 2), whereas type-2X fibers amount to about 7% and 1% in 6J (not shown) and 10ScSn mice (Figure 4A), respectively. Type-2B and -2BX fibers are rare in soleus of both strains (not shown). Adult EDL is composed by a large majority of type-2B/2BX fibers and by about 20% type-2X fibers (Table 2 and Figure 4B), about 6% type-2A fibers (not shown), and occasional presence of type 1 fibers.

Conversely, aging differently affected fiber-type composition between the two strains. In old 6J soleus, only type-2X percentage dropped to 2% (*p* = 0.03; not shown), whereas in old 10ScSn soleus, the percentage of type-1 fibers increased significantly (*p* = 0.05), and that one of type-2A fibers decreased (*p* = 0.04; Table 2), without affecting the amount of type-1/2A (intermediate) and type-2X fiber populations (not shown). Aging did not change fiber-type composition of 6J EDL, whereas it affected 10ScSn EDL, by more than halving type-2X fiber percentage (*p* = 0.001) and increasing type-2B/2BX fibers (*p* = 0.001; Table 2 and Figure 4B). SDH histochemistry, performed to distinguish further among white type-2B and intermediate type-2BX fibers [21,22], revealed that the increase concerned the latter population (45.1 ± 2.7% and 67.1 ± 5.4% for 5 adult and 6 old muscles, respectively, *p* = 0.01; Figure S4), whereas values of white type-2B fibers did not vary. Curcumin treatment affected fiber-type composition only in old 10ScSn EDL, where it significantly counteracted the age-related loss of pure type 2X myofibers (Table 2 and Figure 4B).

Aging did not affect myofiber size of major populations in EDL of both strains, whereas it significantly decreased the size of type-1 myofibers in 10ScSn soleus (−9.25%), and increased that of type-2A of 6J soleus (+25%). Curcumin treatment not only counteracted the aging-induced increase in size of 6J type-2A fibers and the atrophy of 10ScSn type-1 ones, but also increased type-2A fiber size of old 10ScSn soleus (*p* = 0.03 and 0.002, for type-1 and type-2A myofibers, respectively, Table 2).

Myofiber total number did not decrease in soleus and EDL of old 6J mice, whereas it significantly decreased in both muscles (about −16%) of old 10ScSn mice (*p* = 0.03). Curcumin treatment did not affect total myofiber number of soleus, whereas it counteracted the aging-induced loss of EDL myofibers (*p* = 0.03, Table 2).

### 2.4. Effects of Aging and Curcumin Treatment on Costamere Muscle Proteins

Due to the presence of presarcopenic and sarcopenic signatures in old 10ScSn EDL and soleus, respectively, only muscles from this strain were investigated by Western blot analyses focusing on curcumin targets, such as Grp94/gp96 and nNOS [5], and on other costamere proteins and regulators involved in atrophy development [12,23].

**Figure 5:**
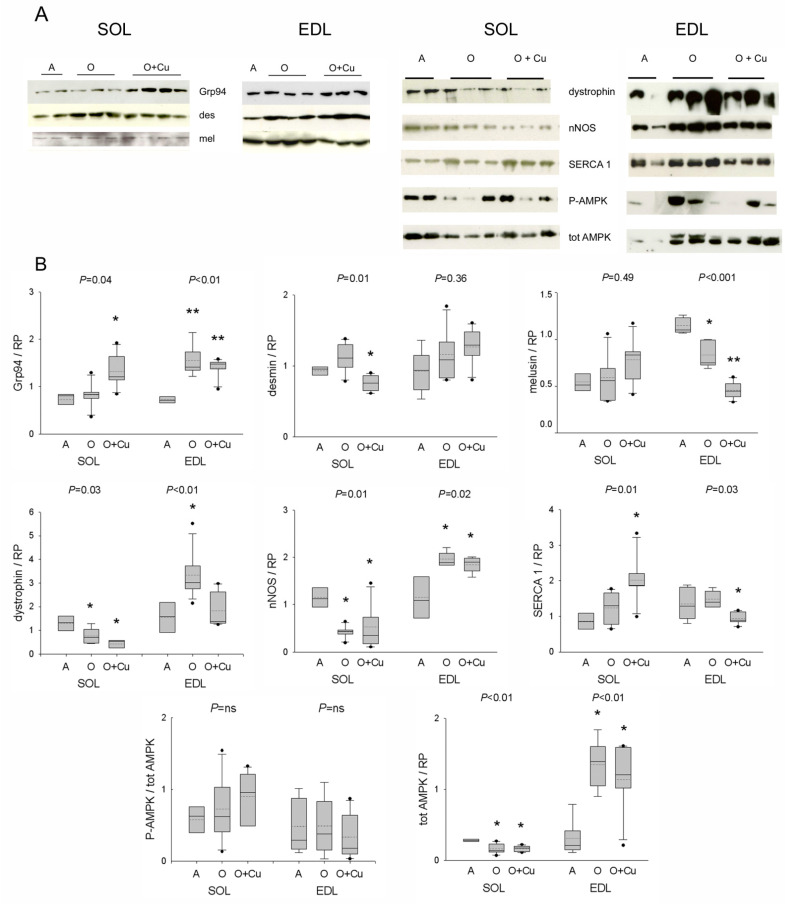
(**A**) Representative Western blots of costameric and regulatory proteins of soleus and EDL of 10ScSn mice. Samples correspond to soleus (SOL) and EDL muscles from Adult mice (A), vehicle-treated Old mice (O) and curcumin-treated Old mice (O + Cu) (see Supplementary Materials for loading reference). (**B**) Box plots of normalized values to total loaded protein stained with Red Ponceau (RP). Soleus and EDL data are expressed in arbitrary units relative to the corresponding adult values. Mean and median values are indicated by dotted and solid lines, respectively. n of soleus or EDL samples was A = 4; O = 5 or 6; O + Cu = 6. Comparisons were performed among groups of the same muscle. Indicated *p* value corresponds to ANOVA test; single or double asterisk indicates the presence of significant difference vs. A, or O, or both, after post-hoc analyses, as detailed in the text.

Aging did not affect soleus levels of Grp94/gp96, desmin and melusin proteins, whereas it strongly reduced both dystrophin and nNOS levels (Figure 5). Curcumin treatment significantly increased Grp94 protein levels, and decreased desmin ones (*p* = 0.01), whereas it did not affect those of melusin, dystrophin and nNOS. Conversely, in the presarcopenic EDL, Grp94, dystrophin and nNOS protein levels significantly increased (*p* ≤ 0.02), whereas those of melusin decreased (*p* = 0.003 vs. adult levels). Curcumin treatment did not change the age-related increase in Grp94 and nNOS levels, nor it affected desmin ones, whereas it blunted the age-related increase of dystrophin protein levels (*p* < 0.01) and reduced further melusin amount (*p* < 0.001).

Protein levels of active and total AMPK were then analyzed, due to the kinase involvement in NO-dependent regulation of myofiber size [12]. Whereas no age- or treatment-related effect was observed in *p*-AMPK/AMPK ratio, total AMPK levels significantly decreased in old soleus and increased in old EDL (*p* ≤ 0.01), and were unaffected by curcumin treatment.

Confocal immunofluorescence confirmed a decreased sarcolemmal presence for both dystrophin and nNOS in old 10ScSn soleus fibers (Figure S5A). However, NADPH-d histochemistry did not reveal an age-related reduction in the sarcolemmal localization of active nNOS (Figure S5B). In addition, it showed an increased frequency of focal subsarcolemmal accumulation (*p* ≤ 0.03; Figure S5B), which was not affected by curcumin. Double staining for nNOS and acetylcholine receptors excluded the association with the neuromuscular junction (Figure S5C). Conversely, the correspondence with SDH-positive foci suggested the co-localization of active nNOS with subsarcolemmal mitochondria (Figure S5C). Labeling with anti-phospho-nNOS antibodies showed the presence of both inhibitory and stimulatory phosphorylation at these sites (Figure S5D). At variance, old EDL muscles showed aggregate-like sarcoplasmic reactivity for nNOS and related inhibitory phosphorylation in a subset of type-2B fibers, consistently with the absence of blue staining with NADPH-d histochemistry (Figure S6A). The possibility that it corresponded to tubular aggregates (TAs) [24] was confirmed by the presence of reactivity for α-sarcoglycan (not shown) and the fast skeletal calcium-pump SERCA1 (Figure S6B). Curcumin treatment strongly reduced TAs in old EDL muscles of both strains (Figure S6, and not shown). Interestingly, curcumin variably affected SERCA1 protein levels, whose amount did not change in pre-sarcopenic and sarcopenic muscles, by increasing them in old soleus, and decreasing them in old EDL (*p* ≤ 0.03; Figure 5).

### 2.5. Effect of Aging and Curcumin Treatment on Myofibrillar Protein Oxidation

Myofibrillar protein oxidation was explored by measuring tropomyosin oxidation, i.e., the formation of disulfide cross-bridge species [25], and presence of carbonylation using Oxyblot [5]. Formation of tropomyosin covalent species appeared detectable only in overexposed films, irrespectively of muscle age and curcumin treatment (Figure S7A), and Oxyblot analysis excluded any age-related increase in myofibrillar protein carbonylation (Figure S7B).

### 2.6. Effects of Curcumin Treatment on Muscle Satellite Cells

**Figure 6:**
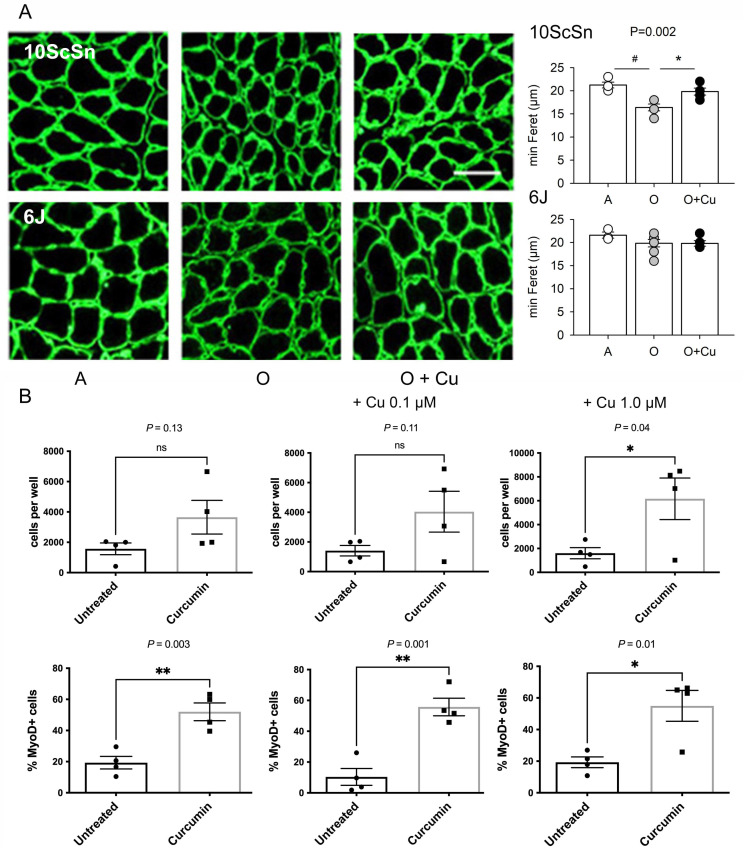
(**A**) Left panels: Representative laminin immunofluorescence of 10-day regenerated soleus myofibers from Adult (A), vehicle-treated old (O) and curcumin-treated old (O + Cu) mice from 10ScSn (top row) and 6J strains (bottom row). Bar: 50 μm. Right panels: Histograms of mean and SEM values of minimal Feret diameter evaluated on at least 400 fibers/muscle from A (n = 4), O (n = 5) and O + Cu (n = 6) 10ScSn soleus muscles and A (n = 3), O (n = 7) and O + Cu (n = 5) 6J ones. Statistical significant difference (ANOVA *p* < 0.002) is indicated by # between A and O *(p* < 0.002), and by * between O and O + Cu (*p* < 0.007). (**B**) Quantification of CD45-/CD31-/Sca1- cells, prepared from hindlimb muscles of curcumin- and vehicle-treated mice, found after 8 days culture in each 0.8 cm^2^ well; 3K cells per well were initially plated at day zero. Top row: total cells, identified by DAPI staining. Bottom row: percentage of MyoD+ cells, identified by immunofluorescence, in the same preps. Statistical significance was calculated using unpaired Student’s *t*-test. Only preps that provided enough cells for MyoD staining are shown.

The percentage of old 10ScSn soleus myofibers displaying central nuclei significantly increased in curcumin-treated muscles compared to vehicle-treated ones (1.25 ± 0.19% and 0.51 ± 0.15%, respectively, n = 6, *p* = 0.01; not shown), suggesting a possible effect of treatment on myofiber regeneration. Therefore, acute damage was induced in adult and old curcumin- and vehicle-treated soleus muscles by injecting notexin [26]. Ten-day regenerated fibers of adult and old 6J muscles did not differ in mean size, whereas those of vehicle-treated old 10ScSn muscles appeared significantly smaller compared to adult (*p* < 0.002) and curcumin-treated ones (*p* < 0.007; Figure 6A).

To investigate whether curcumin treatment affected the behavior of muscle precursors, satellite cells were isolated as CD45-/CD31-/Sca1- from hindlimb muscles of old curcumin- and vehicle-treated 10ScSn mice and placed in growth medium for 8 days (Figure S8). Consistently with literature data [16], their proliferative potential was very low. Culture from curcumin-treated mice yielded on average more cells at day 8, but the difference reached statistical significance only after further in vitro exposure to 1 μM curcumin (Figure 6B and Figure S8). Conversely, the percentage of MyoD-positive cells was significantly higher in cultures obtained after in vivo treatment with curcumin (*p* = 0.003), compared to those of vehicle-treated mice (Figure 6B). In vitro curcumin supplementation did not abolish such a difference (*p* ≤ 0.05, Figure 6B).

## 3. Discussion

This study shows that chronic curcumin systemic administration has general and muscle-specific beneficial effects against aging, without displaying apparent toxicity. Our treatment protocol not only decreased mortality by starting at late adult life (18 mo), at variance with previous evidence [8], but also fully prevented aging-induced muscle mass loss and significantly attenuated force impairment.

Aging variably affected soleus and EDL muscles of two closely related C57BL strains, which were housed in the same animal facility. Based on recently defined criteria [9,10], this study identified sarcopenic signatures in 24-month-old soleus and presarcopenic ones in EDL of 10ScSn mice. Conversely, no mass and/or force loss were detected in soleus and EDL from 24-month-old 6J mice. Curcumin treatment elicited only minor effects in healthy-aged murine muscles, whereas it fully prevented sarcopenia and presarcopenia. Among the possible mechanisms, we provide indications that curcumin acted on the commitment of the satellite cell pool of old muscles.

### 3.1. Strain- and Muscle-Specific Responses to Aging

Genetic background strongly influences lifespan and sarcopenia development [20]. The lack of sarcopenic or presarcopenic signatures in 24-month-old 6J soleus and EDL, examined at a corresponding age of 75 years in humans [19], is consistent with literature reports [19,27]. The presence of sarcopenic and presarcopenic signatures in soleus and EDL, respectively, of the 10ScSn strain might reflect muscle- or fiber-type-specific responses to aging [19,28,29], in addition to genetic differences. Both muscles showed reduction in total myofiber number, but they diverged concerning changes in fiber-type composition and size. Literature on age-related changes in fiber-type composition is controversial, reporting occurrence of a “slower” phenotype or no change at all [19]. Our sarcopenic soleus displayed a fast-to-slow shift accompanying atrophy of type-1 myofibers, consistently with findings in the aging rat [19]. Conversely, the presarcopenic EDL showed a “faster” fiber-type composition, due to the reduction of the type-2X population and the increase of type-2BX one, consistently with observations on rodent tibialis anterior [30,31,32]. Muscle-specificity and fiber-type composition influence also satellite cells distribution [16,20,29], whose abundance significantly declines with age in soleus and EDL muscles [29].

The possibility that contractile performance reflects age-related shifts in fiber-type composition is also a matter of debate [19]. Despite the mass loss, old 10SnSc EDL did not show significant deficits in force development. The prolonged HRTT time cannot be explained by a “faster” fiber-type phenotype. Conversely, old 10ScSn soleus displayed a relevant reduction of absolute force that reflected the loss of muscle mass and the increase in weaker type-1 myofibers. Our data exclude a role for oxidative stress in myofibril derangement, in agreement with comparable findings in old human sarcopenic muscles [14], and isolated myofibers and muscles from old mice and rats [15,17,33,34,35,36].

Sarcopenic and presarcopenic signatures were apparently accompanied by opposite changes in protein levels of costamere interacting molecules, such as dystrophin, nNOS and total AMPK. Reports about age-related changes in dystrophin and nNOS protein levels are controversial [12,37,38,39,40]. Here we show that protein levels of dystrophin, nNOS and total AMPK decreased in the sarcopenic soleus, whereas they increased, together with Grp94/gp96, the chaperone involved in nNOS docking at sarcolemma [5], in the presarcopenic EDL. A large variation in total AMPK amount may affect the actual amount of activated kinase. Reduced AMPK activation has been associated with myofiber atrophy secondary to disuse [40], and aging-induced decrease of nNOS levels to enhanced proteolysis secondary to calpain activation [41]. Furthermore, decreased dystrophin levels may reduce both NO availability and muscle force capacity and render muscle fibers more susceptible to contraction-induced injury [13]. Conversely, the presence of increased protein levels of dystrophin, nNOS and total AMPK in old EDL muscles suggests the availability of a larger amount of NO-dependent activated AMPK that would hamper the appearance of aging-induced myofiber atrophy [39,42].

At variance with disuse atrophy [23], melusin appears dispensable in sarcopenia development, since adult protein levels appeared maintained in 10ScSn sarcopenic soleus, and reduced in old EDL in the absence of myofiber atrophy. The possibility exists that such a muscle-specific, age-related decrease in melusin follows the reduction of FRZB [43], a positive regulator of melusin expression and an inhibitor of the Wnt-signaling pathway [44], which is up-regulated during aging [16].

### 3.2. Curcumin Blunts Presarcopenic and Sarcopenic Signatures

Curcumin administration significantly decreased mortality of aged mice, hampered the loss of muscle mass and attenuated force loss of old soleus. The beneficial effect on survival appeared to occur independently from that on skeletal muscle, since it was also detectable in aging 6J mice, which did not display any apparent muscle involvement yet.

Curcumin treatment prevented the development of soleus sarcopenia in old 10ScSn mice by preserving type-1 myofiber size and inducing hypertrophy in type-2A ones. Such an effect explains the amelioration of the absolute force development and the maintenance of the specific force around adult values, despite the presence of a reduced total myofiber number and a fast-to-slow shift in fiber-type composition. However, we cannot exclude a contribution from the preservation of mitochondrial integrity. Carbonylation of mitochondrial proteins negatively correlated with muscle strength [14] and curcumin was reported to attenuate dicarbonyl species production in mitochondria of COPD dysfunctional soleus muscle [45]. Interestingly, curcumin treatment did not abolish every aging-induced muscle change and scarcely affected costamere proteome, except for Grp94/gp96 protein levels, which appeared up-regulated in the soleus and represented a marker of treatment efficiency, as previously proposed [5]. Treatment did not attenuate the reduction of nNOS, dystrophin and total AMPK protein levels in 10ScSn soleus, mechanistically excluding this regulatory pathway from curcumin anti-sarcopenic action, and suggesting other specific compensatory mechanisms. Here, we provide evidence that curcumin acted on the satellite cell pool of the aging muscle, by increasing its commitment and recruitment into myofibers. Therefore, the curcumin-induced increase in central myonuclei in old soleus is more likely to derive from the addition of new myonuclei via satellite cell activation, to maintain the proper myonuclear domain size as it occurs during muscle hypertrophy [30], than being a consequence of increased myofiber turnover. Curcumin differs from other treatments, such as caloric restriction, or vegetal polyphenols, such as resveratrol, which showed positive effects on lifespan, but detrimental ones at late adult age on satellite cells [20,46]. Apparently, curcumin acted indirectly on muscle precursor commitment, since isolated satellite cells from vehicle-treated old 10ScSn mice did not increase MyoD expression after in vitro exposure to curcumin, at variance with in vivo exposure. Whereas the curcumin effector on muscle precursor commitment remains unknown, Grp94/gp96, which is also up-regulated during muscle differentiation [47], might be among those responsible for the improved maturation of regenerating myofibers in old notexin-injured and curcumin-treated 10ScSn soleus. Our and other laboratories showed both in vitro and in vivo [48,49,50,51] that this chaperone plays crucial roles for differentiation and maturation of muscle precursors, since it is required as the exclusive chaperone for Insulin-like growth factors [49] and a mTOR regulator [51].

Curcumin treatment prevented also presarcopenia, as we showed for EDL of old 10ScSn mice. In this case, loss of muscle mass was secondary only to the decrease in total myofiber number, and curcumin administration blunted myofiber loss by concomitantly preserving type 2X myofiber population, which represents about 20% of total fibers in the adult EDL. The selective aging-induced reduction of this population is suggestive of denervation events [30]. Repeated denervation/reinnervation leads also to the increase of type-2BX fibers within the same motor unit [30]. The beneficial effects of curcumin on satellite cells might antagonize reinnervation failure and loss of myofibers. In fact, satellite cells appear to be required for the integrity of the neuromuscular junction in EDL [52]. In addition, curcumin might exert cytoprotective effects on 2X motoneurons or their afferences, as suggested by evidence obtained both on neurons and peripheral nerves [53,54,55]. The rather low number of type-2X myofibers in the soleus muscle may tentatively explain the lack of curcumin effect on the preservation of soleus total myofiber number.

Although we cannot exclude a muscle-specific response to our treatment, chronic systemic curcumin administration appears to be very efficient in aging mice, since it significantly decreased spontaneous mortality during senescence, efficiently counteracted presarcopenia and greatly attenuated sarcopenia, improving satellite cell commitment and recruitment. In addition, the low frequency of subcutaneous administration of our curcumin formulation, compared to the use of osmotic pumps [17], represents a major strength toward a future translatability of this protocol to humans. Present therapeutic strategies aim to counteract senescence through the ability of different molecules to disrupt pro-survival pathways active in senescent cells (senolytic compounds) or to hamper the development/release of dysfunctional signals (senostatic compounds) [30,56,57]. Curcumin may exert both types of action, due to its pleiotropic and hormetic properties [58]. However, the effector and the nature, senolytic or senostatic, of the positive influence of curcumin on muscle cell precursors remains to be determined.

## 4. Materials and Methods

### 4.1. Curcumin Formulation

A 100 μL volume of 50 mg/mL curcumin in ethanol was added to 1.5 mL of 100 mg/mL hydroxypropyl-β-cyclodextrins (HPβCD) in 0.15 M NaCl, 0.2 mM phosphate buffer (PBS) pH 7.4. The mixture was stirred at room temperature (RT) for 72 h in the dark and then ethanol was eliminated under mild vacuum. The dispersion was centrifuged at 4000 rpm for 20 min and the curcumin content in the solution was assessed by RP-HPLC using a C18 column (Luna-C18 column 5 μm, 250 × 4.6 mm, Phenomenex) isocratically eluted with a 48:52% *v*/*v* of 0.5% *w*/*v* citric acid (pH 3.0)/acetonitrile mixture at 1 mL/min flow rate. The UV detector was set at 429 nm. Curcumin concentration in the solution was calculated on the bases of the titration curve obtained with 1–10 μg/mL solutions of curcumin in PBS:y (peak area) = 144,828 × (curcumin concentration) + 12,628, R^2^ 0.999. The curcumin solution was lyophilized and then the solid was reconstituted with 1.5 mL water and analyzed by RP-HPLC as reported above. Curcumin solutions were maintained in the dark at −20 °C until used.

### 4.2. Mice

Founders from 6J (C57BL/6J) and 10ScSn (C57BL/10ScSn/OlaHsd) mouse strains were obtained from Jackson laboratories and Envigo, respectively. Mice were bred in the same animal house facility. Six-month-old male mice (n = 30; 15 from each strain) were used without any treatment. Eighteen-month-old mice (n = 113, Table 1) were injected subcutaneously, every sixth day, for 6 months, with about 120 μg/kg of the curcumin formulation in a volume of 100 μL, or an equal volume of HPβCD. Events of spontaneous death during treatment were registered. The surviving mice (36 from 6J strain and 41 from 10ScSn) were used in the study. From these, 25 mice from 6J strain and 29 from 10ScSn one were euthanized at 24 months of age together with 6-month-old ones (n = 23 from both strains). Soleus and EDL muscles were excised bilaterally and either frozen in liquid nitrogen and stored at −80 °C for immunohistochemistry and Western blotting (n = 13 from 6J mice and n = 17 from 10ScSn mice) or immediately processed for mechanical studies (n = 18 from 6J mice and n = 21 from 10ScSn mice). Liver samples were also collected and frozen in liquid nitrogen. Hind limb muscles were excised from 24-old-month 10ScSn mice for satellite cell isolation.

Acute degeneration was induced in the left soleus of adult and 24-month-old mice (n = 15 for each strain), by injecting 75 μL of the myotoxic drug notexin (0.5%, Sigma) under general anesthesia [26]. Mice were euthanized 10 days after surgery, and muscles excised and frozen in liquid nitrogen for subsequent studies.

### 4.3. Mechanical Recordings

Freshly isolated soleus and EDL muscles were analyzed using a vertical muscle apparatus (300B, Aurora Scientific Inc, Bristol, UK) containing a Ringer solution at 30 °C bubbled with 95%O_2_–5%CO_2_ [26,59]. Muscles were stretched to the optimal length and electrically stimulated, by two parallel electrodes, with supramaximal pulses (0.5ms duration) delivered by a Grass S44 electronic stimulator through a Grass stimulus isolation unit. Muscle response was recorded through a Grass isometric force transducer connected to an AT-MIO 16AD acquisition card (National Instruments) [26,59]. Time to peak of the twitch (CT), half relaxation time of the twitch (HRT) and of the tetanus (HRTT), and the maximum rate of rise of tetanic tension (Vmax) were measured. Twitch and tetanic tensions were normalized to the muscle wet weight (specific tension, Nxg-1). Force-frequency curve was determined by stimulating soleus muscle at 1, 20, 40, 60, 80, 100, 120 and 150 Hz and EDL muscle at 1, 30, 45, 60, 75, 90, 120, and 150 Hz. Muscles were weighed at the end of each experiment.

### 4.4. Antibodies and Reagents

The following antibodies were used. Anti-Phospho-AMPK rabbit monoclonal Antibody (mAb), anti-AMPK and anti-cleaved Caspase-3 rabbit polyclonal antibodies (pAb) were from Cell Signaling. Anti-desmin (clone DE-U-10) mouse mAb, and anti-melusin (ITGB1BP2) and anti-laminin rabbit pAbs were from Sigma. Anti-nNOS phospho-S1417 and -S847, anti-dystrophin and anti-Grp94 rabbit pAbs were from Abcam. Anti-NOS1 and anti-CHOP rabbit pAbs, anti-SERCA-1 goat pAb, anti-SERCA-2 and anti-tropomyosin mouse mAbs were from Santa Cruz Biotech. Anti-Ki67 (clone MM1), anti-MyoD (clone 5.8A) and anti-α-sarcoglycan (clone Ad1/20A6) mouse mAbs were from Novocastra, BD Pharmingen and Monosan, respectively. Anti-Grp94 (clone 3C4) mouse mAb ([47] and Millipore) and anti-myosin heavy chain (my) mouse Abs anti-1 (clone BA-D5), anti-2A (clone SC-71) and anti-all types except 2X (clone BF-35) [21] were previously described.

Secondary antibodies conjugated with peroxidase were from Cell Signaling and Dako Cytomation, and those conjugated with AlexaFluor-488 or AlexaFluor-568 were from Invitrogen.

FITC-conjugated α-bungarotoxin was from Invitrogen.

### 4.5. Routine Histology, Immunofluorescence and Immunoperoxidase

Liver and transverse muscle cryosections (10 μm-thick) were collected on gelatin-coated slides and processed for haematoxylin-eosin staining, indirect immunofluorescence or immunoperoxidase. Processing was as previously described [21,25,26,47], except for the following antibodies:

(1) labeling of Ki67 required fixation with 4% buffered paraformaldehyde (PFA) for 20 min at RT and epitope unmasking by two-times 5 min-boiling in citrate buffer pH 6.0;
(2) labeling of activated-caspase 3 required fixation with 3% PFA for 15 min at RT;
(3) labeling for dystrophin required fixation with 4% PFA for 10 min at RT;
(4) labeling for phospho-nNOS-S847 and -S1417 required fixation with 4% PFA for 5 and 15 min, respectively;
(5) double labeling for SERCA1 and SERCA2 required fixation with 4% PFA for 10 min. Incubation with antibodies was performed in 5% donkey serum.

Imaging was performed by either optic microscopes, equipped with epifluorescence (Leica RD100 or Axioplan, Zeiss), or by confocal microscopy (Leica SP5). Morphometric measurements were done using the ImageJ NIH software. Ki67-positive hepatocyte nuclei were counted on whole cryosections; the percentage of CHOP-positive nuclei was evaluated on more than 600 hepatocytes from three randomly selected fields. Whole muscle transverse cryosections were used for total myofiber count and fiber-type quantification. Myofiber size was determined by measuring the minimal Feret diameter [23], on at least 60 fibers for each fiber-type from two different micrographic fields of the same muscle. In regenerating muscle, minimal Feret diameter was measured at least in 400 regenerated fibers in the mid-belly region [26].

### 4.6. SDH and NADPH-Diaphorase (NADPH-d) Histochemistry

Histochemistry for SDH and NADPH-d was performed on soleus and EDL cryosections following previously described protocols [5,21]. Morphometric analyses were performed on micrographs independently by two investigators using ImageJ. The percentage of NADPH-d-positive sarcolemma was calculated on myofiber cross-sectional circumference (CSC; [5,25]).

### 4.7. Western Blotting

A small fragment of soleus and EDL muscle was cut and homogenized by repeated pipetting in 100 μL of hot Laemmli 2× buffer. After protein determination [5], equal amounts of proteins were separated by polyacrylamide gel electrophoresis (PAGE) in reducing and denaturing 4–13.5% gradient gels, and transferred to nitrocellulose in the presence of methanol. Blot strips were incubated, after blocking, with appropriate dilutions of primary and secondary antibodies. Bound antibodies were revealed using chemiluminescence, as previously described [23]. Relative protein levels were calculated by normalization of the densitometric signal to the corresponding amount of total loaded proteins, after staining with Ponceau Red.

### 4.8. Tropomyosin Oxidation and Oxyblot

Disulfide cross-bridge formation in tropomyosin was investigated by separating SH-blocked crude myofibrillar preparations in both non-reducing and reducing PAGE, followed by immunostaining with the anti-tropomyosin antibody [25]. Protein carbonyl groups were demonstrated using the OxyBlot protein oxidation detection kit (Millipore), as previously described [5]. The degree of protein carbonylation was determined after normalization to loaded protein.

### 4.9. Satellite Cell Isolation and Culture

Hind limb muscles were homogenized in DMEM (Gibco) using a Miltenyi gentleMACS dissociator. Upon decanting, triturated muscle was digested in Collagenase A (Sigma) and DNAse I (Roche) in DMEM, filtered through 80 μm and 20 μm nylon mesh and pelleted. Cells were resuspended in PBS containing also 0.5% bovine serum albumin 2 mM EDTA, and incubated with CD31 and CD45 microbeads (Miltenyi) before being subjected to magnetic separation in MACS columns. The unretained CD31^-^CD45^-^ fraction was then incubated with Sca-1-microbeads and processed as described above. After quantification with DAPI and propidium iodide, CD31^-^CD45^-^Sca-1 cells were seeded in 8-well chamber slides (LabTek) at 3000 live cells/well in F12-Ham medium supplemented with 1% penicillin/streptomycin, 20% Fetal Calf Serum (Gibco) and 5 ng/mL murine FGFb. Cells were cultured for eight days in the presence or in the absence of curcumin.

### 4.10. Statistical Analyses

The experimental unit corresponded to the single mouse. Data were expressed as mean, median, and 5th–95th percentiles with outliers, when using dot or box-plot representation, and as mean ± SEM, when using histograms. Statistical analysis for survival between 18–24 months of age was analyzed using log-rank (Mantel-Cox) test. Data were analyzed using the one-way analysis of variance (ANOVA) followed by Newman-Keuls post hoc test. Unpaired Student’s *t*-test was used when comparing two groups. *p* values less than or equal to 0.05 were considered statistically significant. Analyses were performed using Statistical Package SigmaStat version 2.0 (Jandel Europe, Germany).

## Figures and Tables

**Table 1:** Body and hindlimb muscle weights of adult and old mice from two C57BL strains.

C57BL Strain	Age (mo)	Curcumin Treatment (6 mo)	BW (g)	Soleus Weight (mg)	Soleus MW/BW	EDL Weight (mg)	EDL MW/BW
**6J**							
(n = 8)	6	-	30.9 ± 1.3	8.9 ± 0.5	0.28 ± 0.01	10.0 ± 0.5	0.34 ± 0.02
(n = 10)	24	-	33.1 ± 0.6	8.6 ± 0.4	0.25 ± 0.01	11.4 ± 0.4	0.34 ± 0.01
(n = 8)	24	+	32.9 ± 0.5	8.7 ± 0.3	0.26 ± 0.01	11.8 ± 0.7	0.34 ± 0.02
**10ScSn**							
(n = 9)	6	-	31.8 ± 1.5	8.3 ± 0.1	0.28 ± 0.01	10.3 ± 0.3	0.30 ± 0.01
(n = 8)	24	-	35.4 ± 1.6	6.9 ± 0.5	0.19 ± 0.01 **	9.0 ± 0.2 *	0.25 ± 0.01 *
(n = 9)	24	+	32.4 ± 0.5	8.1 ± 0.5	0.24 ± 0.01	9.7 ± 0.3	0.29 ± 0.01

NOVA ** ≤ 0.01 vs. all; * ≤ 0.04 vs. all.

**Table 2:** Percentage, size and total number of soleus and EDL myofibers of adult and old mice of two C57BL strains.

C57BL Strain (n Animal)	Age (mo)	Curcumin Treatment (6 mo)	Major Fiber Type Percentage		Minimal Feret’s Diameter (m)		Myofiber Total Number
**Soleus**			**Type 1**	**Type 2A**	**Type 1**	**Type 2A**	
**6J**							
(n = 5)	6	-	38.8 ± 3.3	54.2 ± 4.2	38.4 ± 1.5	35.7 ± 2.4	887.2 ± 65.7
(n = 4)	24	-	32.0 ± 1.6	65.6 ± 1.5	49.8 ± 5.6	44.2 ± 2.4 *^ac^	796.2 ± 137.9
(n = 4)	24	+	41.6 ± 3.6	56.3 ± 3.6	42.0 ± 2.5	39.8 ± 2.1	765.0 ± 64.3
**10ScSn**							
(n = 6)	6	-	37.5 ± 2.7	56.8 ± 1.7	40.2 ± 1.6	36.1 ± 1.0	1067.6 ± 30.5
(n = 6)	24	-	47.9 ± 3.8 *^a^	47.8 ± 3.5 *^a^	35.7 ± 0.7 *^ac^	35.5 ± 0.6	905.3 ± 47.3 *^a^
(n = 5)	24	+	52.7 ± 2.8 *^a^	46.3 ± 3.0 *^a^	40.9 ± 0.7	40.8 ± 0.9 **	872.0 ± 48.5 *^a^
**EDL**			**Type 2X**	**Type 2B/2BX**	**Type 2X**	**Type 2B/2BX**	
**6J**							
(n = 5)	6	-	17.5 ± 2.4	74.1 ± 3.8	29.7 ± 2.2	37.6 ± 2.5	962.2 ± 49.8
(n = 4)	24	-	18.6 ± 3.9	72.5 ± 3.4	28.9 ± 1.6	36.2 ± 1.6	841.0 ± 38.1
(n = 4)	24	+	16.2 ± 4.2	72.6 ± 6.7	32.8 ± 0.9	40.1 ± 1.1	893.2 ± 19.5
**10ScSn**							
(n = 5)	6	-	21.8 ± 1.6	72.6 ± 2.3	26.4 ± 1.8	39.2 ± 2.4	999.2 ± 72.7.
(n = 6)	24	-	9.3 ± 1.9 **	85.6 ± 1.9 **	28.6 ± 2.8	36.8 ± 1.7	826.8 ± 30.0 *^ac^
(n = 5)	24	+	20.4 ± 2.3	77.1 ± 2.2	26.9 ± 1.0	34.8 ± 1.7	927.8 ± 32.2

Values correspond to mean and SEM. ANOVA and post-hoc: *^a^ *p* ≤ 0.05 vs. adult; *^ac^ *p* ≤ 0.05 vs. all; ** *p* < 0.01 vs. all.

## Data Availability

not applicable.

## Supplementary Materials

The following are available online at https://www.mdpi.com/article/10.3390/ijms222111789/s1.
