# Supplementary material for: Chronic Systemic Curcumin Administration Antagonizes Murine Sarcopenia and Presarcopenia
Source: Int J Mol Sci. 2021 Oct 30;22(21):11789. doi: 10.3390/ijms222111789 (PMC8584127; doi:10.3390/ijms222111789)
Supplement: Supplementary file 1 [file ijms-22-11789-s001.zip › ijms-1428033-supplementary.pdf]

Supplemental Figures and Western blot data

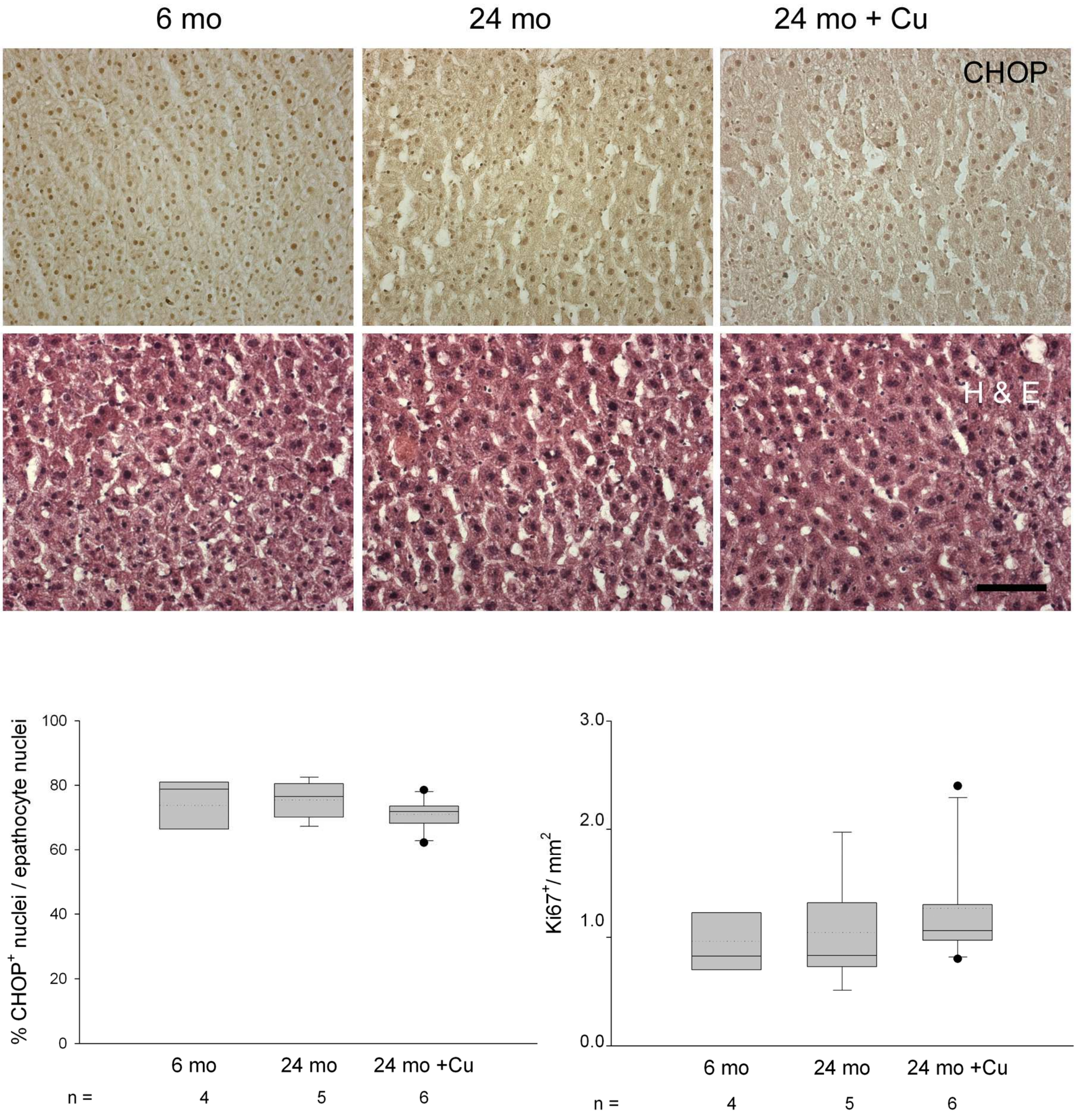

Figure S1: **Liver effects of curcumin treatment.**

A) Representative micrographs of liver cryosections from adult 10ScSn mice (6mo) and old ones, after either vehicle (24mo) or Curcumin administration (24mo+Cu), processed for immunoperoxidase with antibodies for the ER-stress inducible transcription factor CHOP (upper row) or for routine haematoxylin and eosin (H&E). Bar: 150µm.

B) Box plots illustrate the percentage of CHOP-positive hepatocyte nuclei to total hepatocyte nuclei evaluated on about 1000 hepatocytes from different regions in each sample. n indicates the number of examined samples. Median and mean values correspond to solid and dotted line in the box, respectively.

C) Box plots show the density of hepatocytes dysplaying positive nuclei for the proliferation marker Ki67. Examined liver cryosections were larger than 0.5 cm^2.

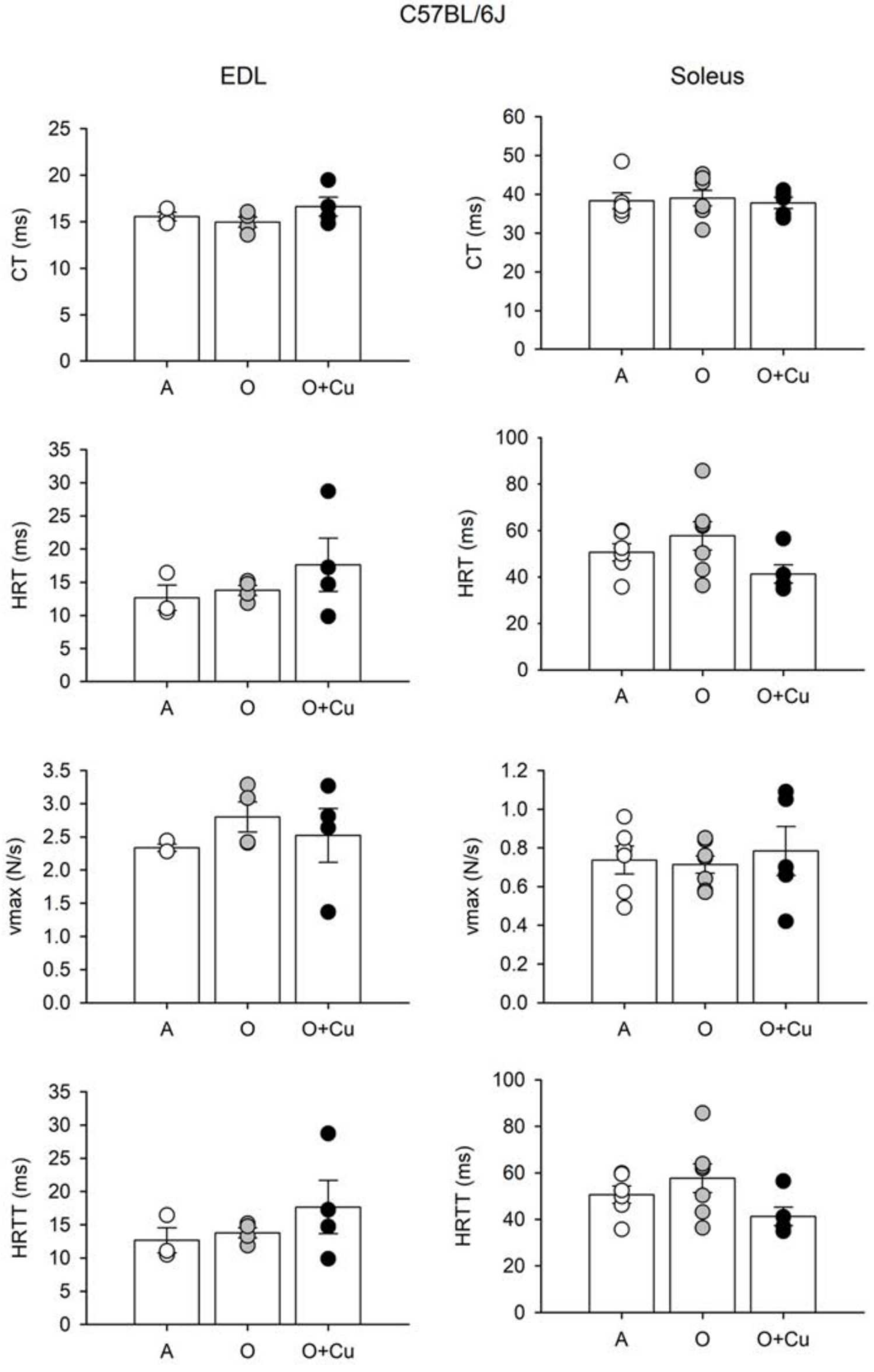

Figure S2: **Contractile parameters of adult, old and old-treated soleus and EDL muscle of C57BL6J mice.**

Histograms represent: contraction time (CT), half relaxation time of the twitch (HRT), maximum rate of rise of the tetanus (Vmax) and half relaxation time of the tetanus (HRTT) for EDL and soleus from Adult 6J mice (A) and old ones, after either vehicle (O) or Curcumin administration (O+Cu). Values are mean ± SEM. For EDL, A, n=3; O, n=3, O+Cu, n=4; for soleus, A, n= 6; O, n= 7; O+Cu, n=5.

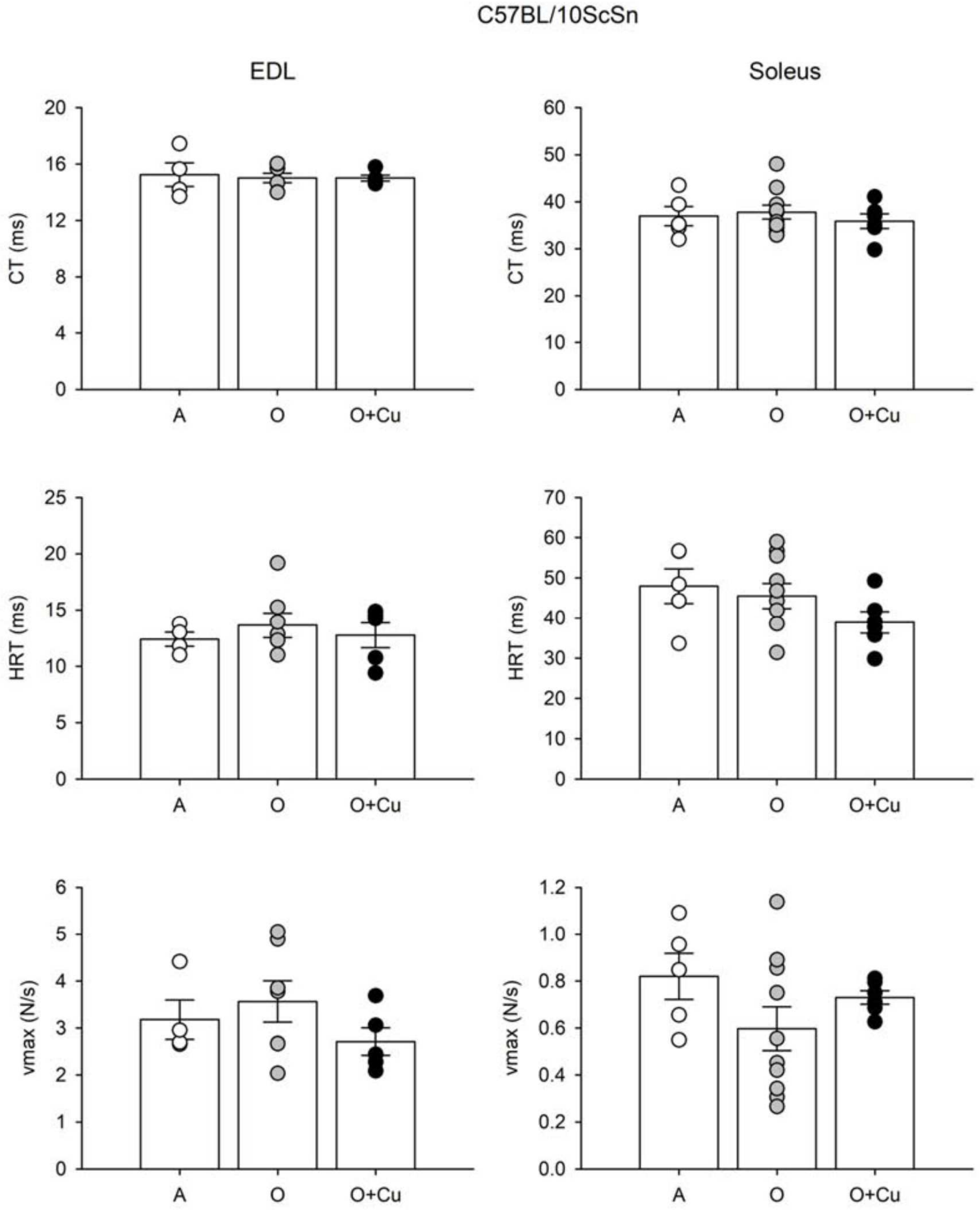

Figure S3. **Contractile parameters of adult, old and old-treated soleus and EDL muscle of C57BL10ScSn mice.**

Histograms represent: contraction time (CT), half relaxation time of the twitch (HRT) and maximum rate of rise of the tetanus (Vmax) for EDL and soleus from Adult 10ScSn mice (A) and old ones, after either vehicle (O) or Curcumin administration (O+Cu). Values are mean± SEM. For EDL, A, n=4; O, n=7, O+Cu, n=5; for soleus, A, n=5; O, n=10; O+Cu, n= 6.

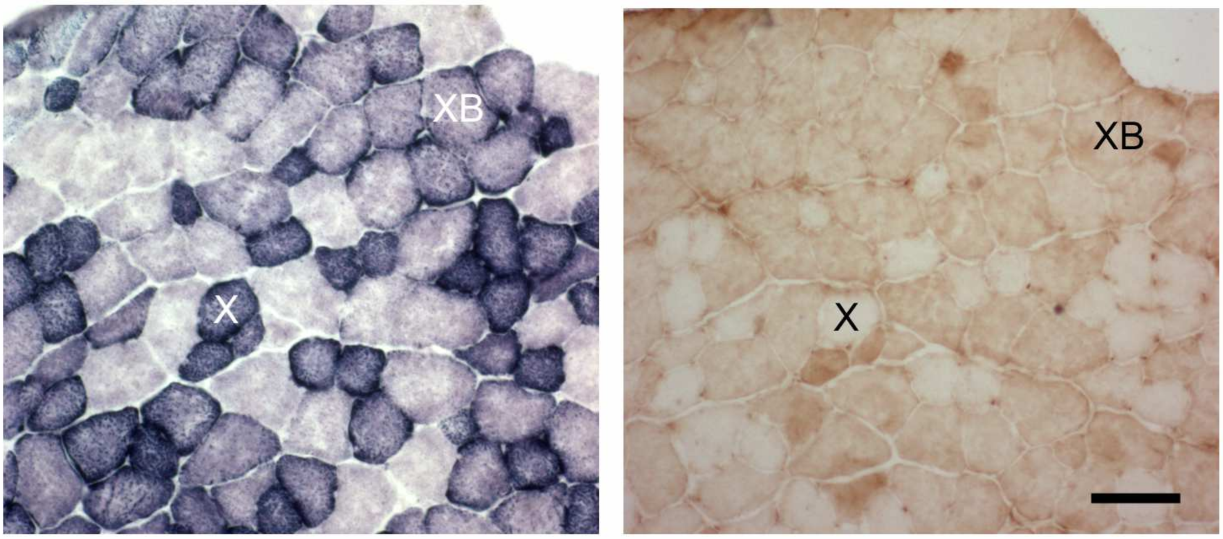

Figure S4: SDH histochemistry of old C57BL10ScSn EDL muscle.

Representative serial cryosections from an old 10ScSn EDL muscle stained for succinate dehydrogenase (SDH) histochemistry (left panel) and immunoperoxidase after staining for all myosins except my-2X (BF-35 antibody; right panel). Positive staining for SHD is detected in small myofibers (X) and in large myofibers (2BX). Absence of staining with anti-myosin antibody BF-35 identifies X as type-2X fibers. Presence of antimyosin staining occurs in both type-2BX fibers and in SDH-white type-2B ones. Bar:50μm.

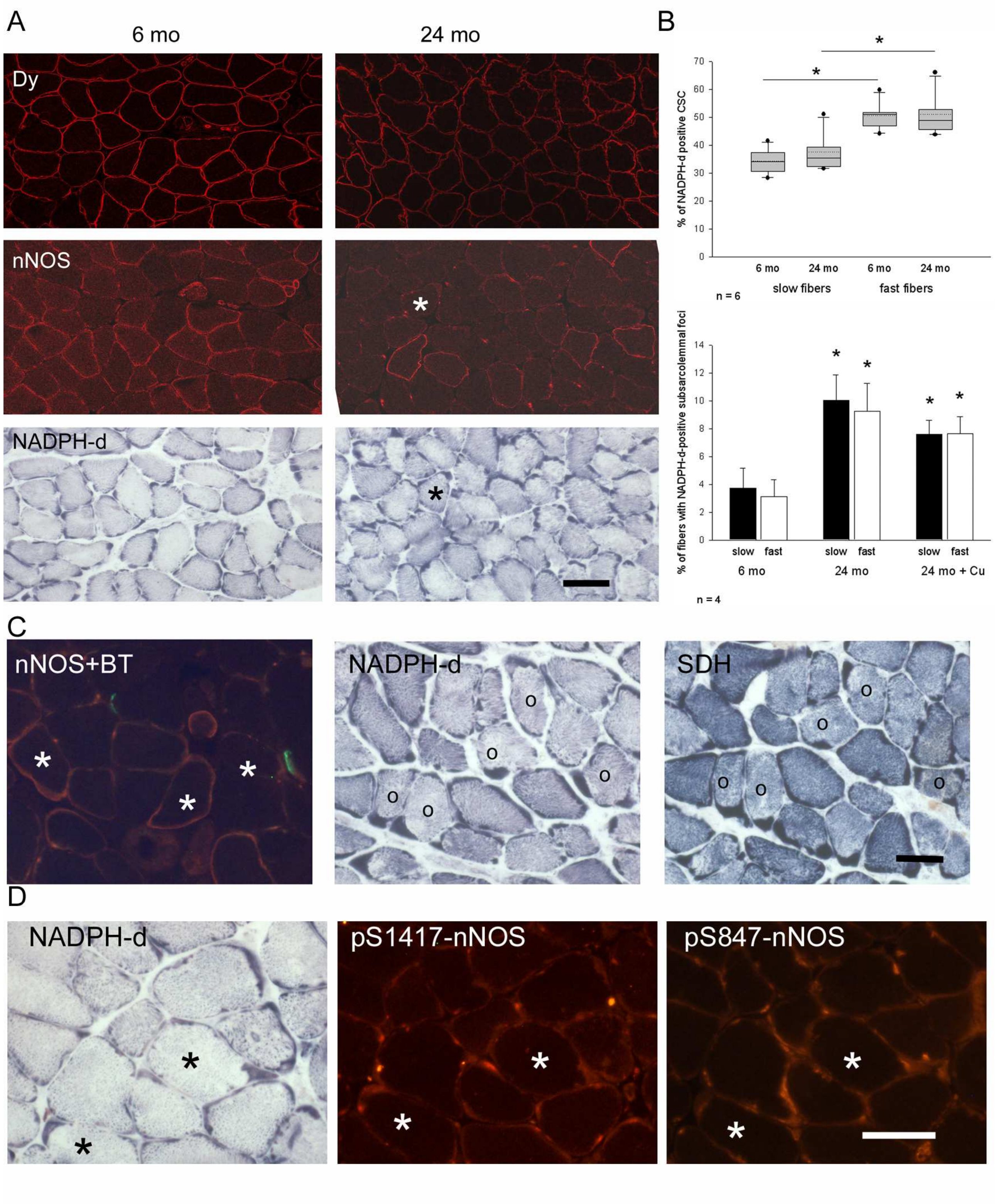

Figure S5: **Distribution of dystrophin and nNOS immunoreactivity and NADPH-d histochemistry in adult and old C57BL10ScSn soleus muscles.**

A) Representative micrographs of adult (6 mo) and old (24 mo) soleus from the 10ScSn mouse strain, processed for confocal microscopy for dystrophin (upper row) and nNOS (middle row) immunofluorescence and for NADPH-d histochemistry (lower row). Asterisk indicates a fiber with subsarcolemmal accumulation of active nNOS. Bar: 100µm.

B) Upper panel: Box plots of percentage of myofiber cross-sectional circumference (CSC) positive for NADPH-d histochemistry in adult and old 10ScSn soleus muscles. Median and mean values are

indicated with a solid and dotted line, respectively. ANOVA P=0.03. Asterisks indicate presence of significant difference between values of slow and fast fibers of either adult or old muscles. N indicates number of animals studied. Lower panel: Histograms of the percentage of positive foci for NADPH-d detected at sarcolemma of slow and fast fibers from soleus of adult, old and old-treated 10ScSn mice. ANOVA P=0.03. Asterisks indicate presence of significant difference between values of slow and fast fibers of either adult or old muscles. N indicates number of animals studied.

C) Representative micrographs of 24-mo-old 10ScSn soleus processed for immunofluorescence microscopy (left panel) for nNOS (red fluorescence) and a-Bungarotoxin (green fluorescence) and for histochemistry for NADPH-d activity (middle panel) and SDH one (right panel). Myofibers showing discrete accumulation of nNOS immunoreactivity at sarcolemma are indicated by asterisks, whereas void circles were used to identify the presence of subsarcolemmal foci of NADPH-d and SDH activity in the same myofibers. Bar: 50μm

D) Representative serial micrographs of 24-mo-old 10ScSn soleus processed for NADPH-d histochemistry (left panel) and for immunofluorescence microscopy (middle and right panel) with two phospho S-nNOS antibodies (middle and right panels). Asterisks indicate myofibers showing the presence of subsarcolemmal foci of NADPH-activity and immunofluorescence for both stimulatory (pS1417) and inhibitory (pS847) nNOS Ser-phosphorylation. Bar: 50μm

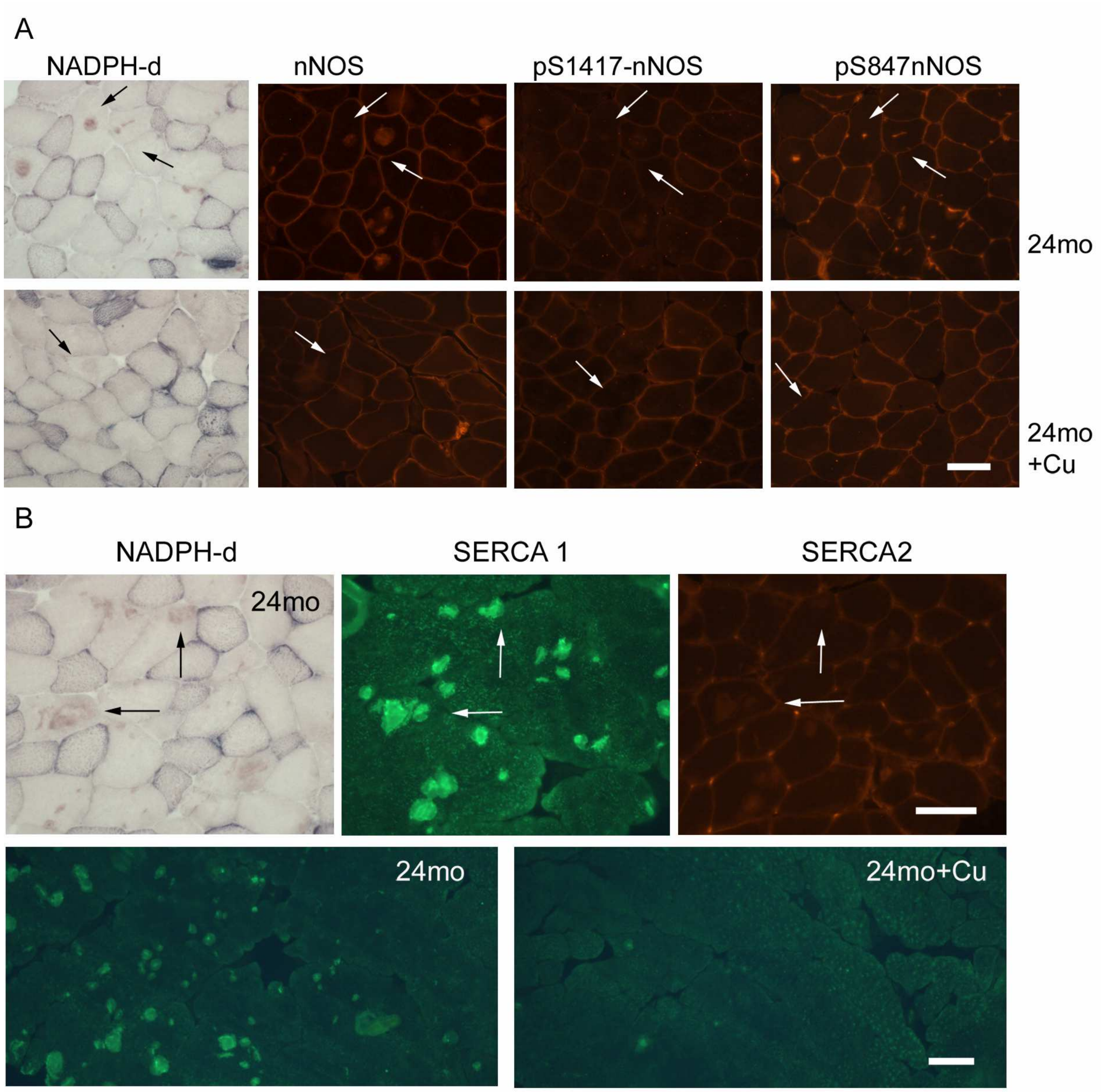

Figure S6: Distribution of nNOS and SERCA1 immunoreactivity, and NADPH-d histochemistry in old and old-treated C57BL10ScSn EDL muscles.

A) Representative micrographs from 24 mo-old 10ScSn EDL muscles from mice injected with vehicle (24mo) or curcumin (24mo+Cu), were obtained from serial cryosections processed for NADPH-d histochemistry (left panel) and indirect immunofluorescence for nNOS (middle panel) and phospho-nNOS (pS1417 and pS847; right panels). Arrows indicate large myoplasmic accumulation of nNOS, which displays abnormal red NADPH-d reactivity and positive inhibitory Ser-phosphorylation and disappear from myofibers of curcumin-treated mice. Bar: 50μm.

B) Upper row: Representative cryosections of 24 mo-old 10ScSn EDL processed for NADPH-d histochemistry (left panel) and double immunofluorescence for SERCA1 (middle panel) and SERCA2 (right panels). Arrows indicate large myoplasmic accumulation of SERCA1, which correspond to regions with abnormal red NADPH-d reactivity. Bar: 50μm Lower row: Representative low magnification fields of EDL

muscle obtained from 10ScSn mice injected with veicle (24mo) or curcumin (24mo+Cu) and stained with indirect immunofluorescence for SERCA1. Bar: 100µm.

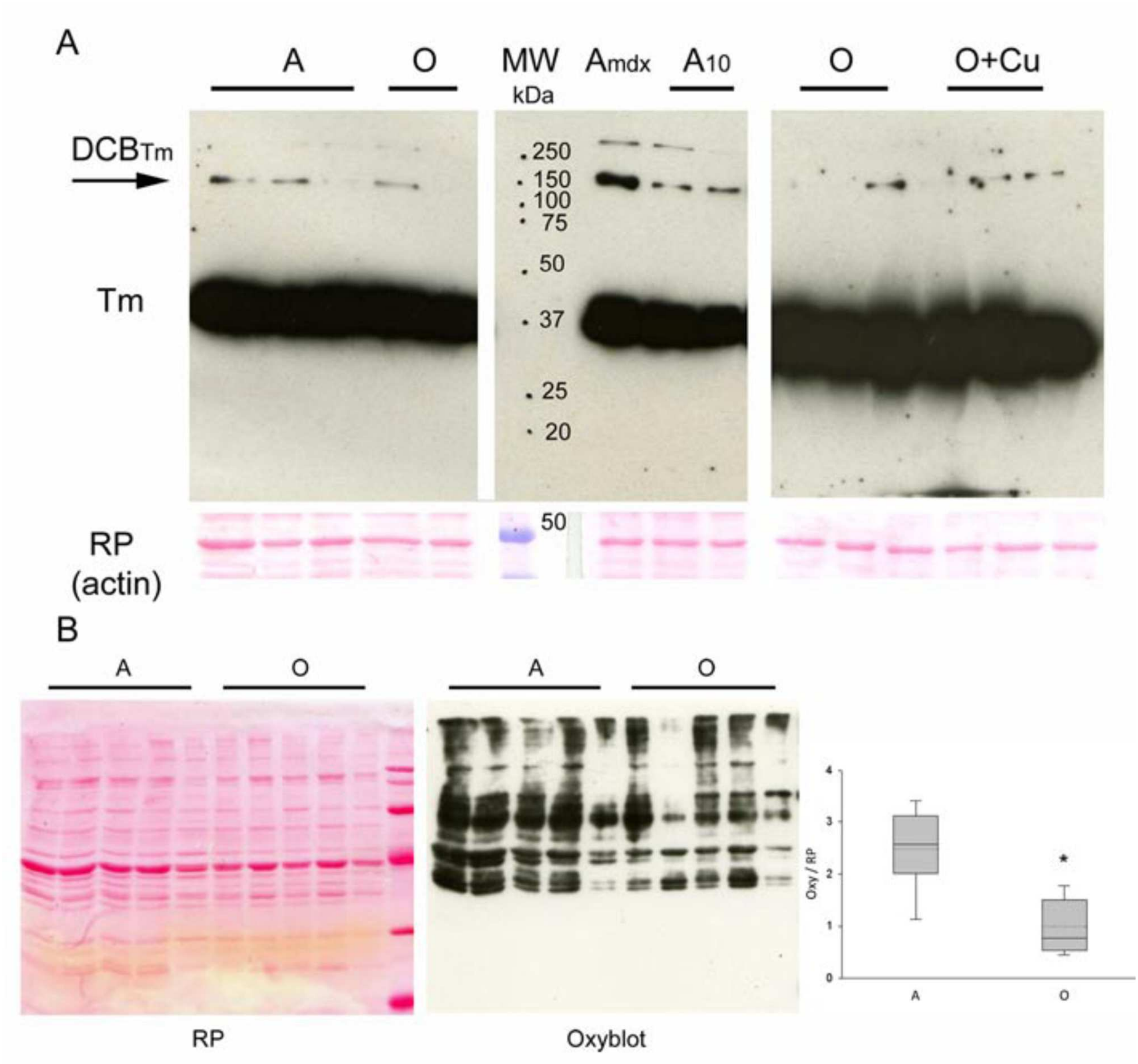

Figure S7: **Carbonylated proteins (Oxyblot) and tropomyosin covalent species in C57BL10ScSn hindlimb muscles.**

A) Representative Western blots of non-reducing SDS-PAGE gels stained for tropomyosin (Tm). Lanes were loaded with crude myofibrillar preparation obtained from the soleus muscle of adult mice (A), vehicle-treated old (O) and curcumin-treated old (O+Cu) mice of the 10ScSn strain, and from adult diaphragm of dystrophic (A mdx) and 10ScSn (A10) mice. Arrow indicates the presence of Tm-derived disulfide cross-bridge species (DCB-Tm). Left and middle panels correspond to parallel gels exposed to the same autoradiographic film. Note the stronger signal corresponding to DCB-Tm in the Amdx sample. Lower panels show loading of the corresponding lane by staining of actin with Red Ponceau (RP).
B) Left and middle panels show RP and Oxyblot stainings of myofibrillar lysates from A and O 10ScSn gastrocnemius muscle. Right panel shows box plots of Oxyblot densitometric values normalized to RP. Mean and median values are indicated by dotted and solid lines, respectively. n=5. Asterisk indicates significant difference between A and O values (P= 0.04, Student's t test).

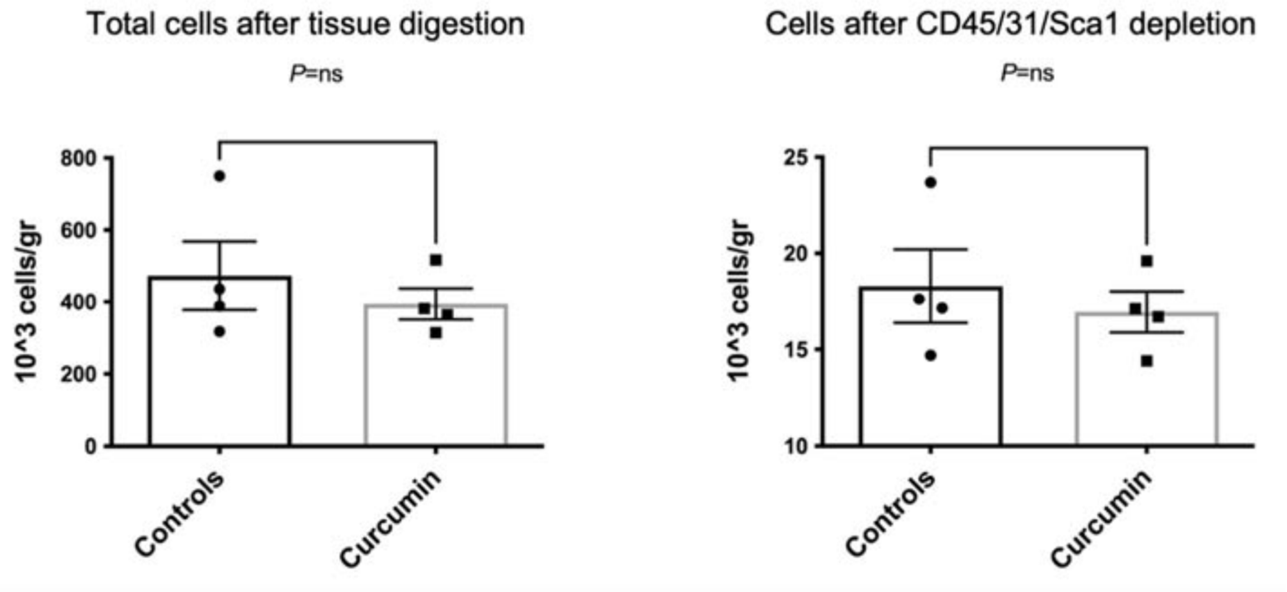

Figure S8: **Total amounts of mononucleated cells isolated from old and old-treated C57BL10ScSn hindlimb muscles**

Left chart: number of total mononucleated cells obtained from digestions of hindlimb muscles of treated and untreated (controls) animals. Right chart: number of mononucleated cells obtained from the same preps upon depletion for CD45/CD31/Sca1 with immunobeads. Statistical analyses were performed with Student's t-test.

Original Western blots:

Figure 5 : Soleus muscle

Red Ponceau WB 1

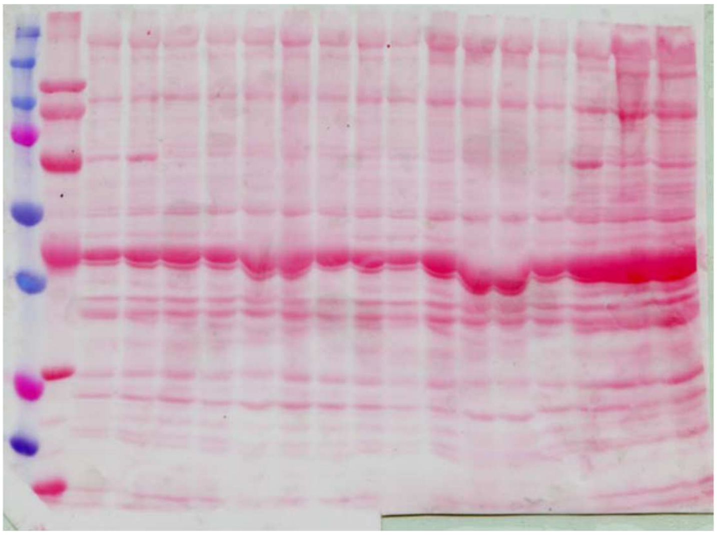

Loading:
1.MW
2.MW
3. Adult
4. Adult
5. Old
6.Old
7.Old
8. Old+Cu
9. Old+Cu
10. Old+Cu
11. Old+Cu
12. Old+Cu
13. Old+Cu
14. Old
15.Old
16. Adult
17.Adult
18. Old

Anti-Grp94 WB1

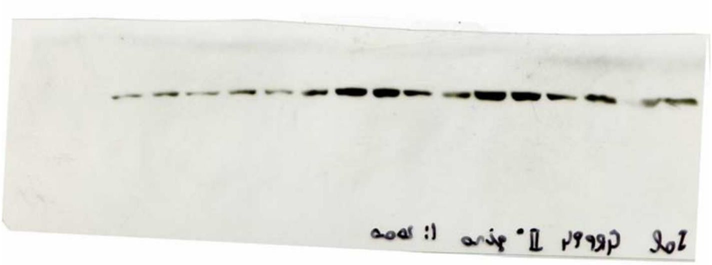

Anti-desmin WB1

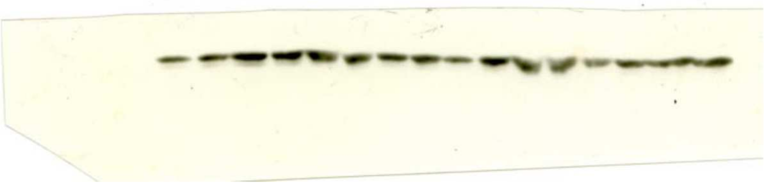

Figure 5 : Soleus muscle
Red Ponceau WB 2

Loading:
1. Adult
2. Adult
3. Adult
4. Adult
5. Old
6.Old
7.Old
8. Old+Cu
9. Old+Cu
10. Old+Cu
11. Old
12. Old
13. MW

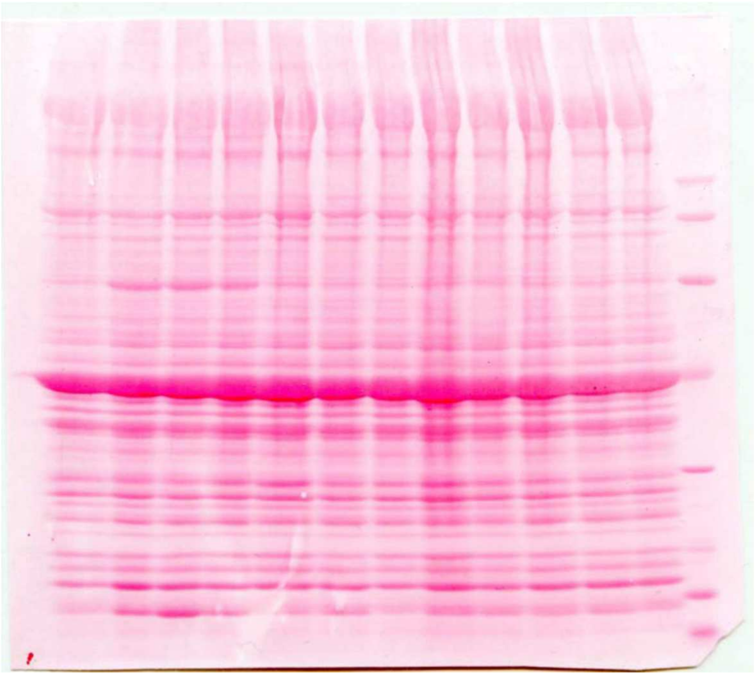

Anti-melusin WB2

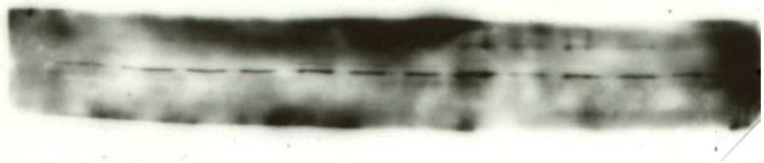

Anti-dystrophin WB2

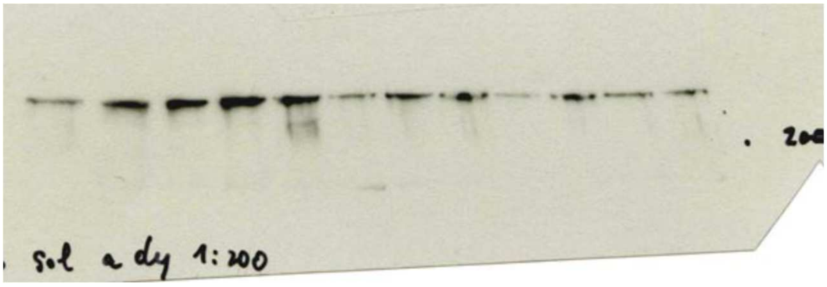

Anti -nNOS WB2

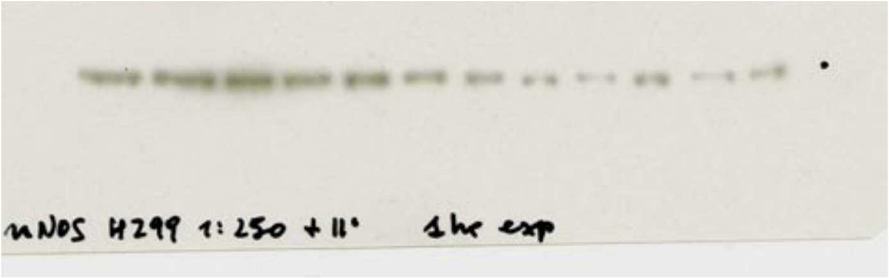

Anti-SERCA1 WB2

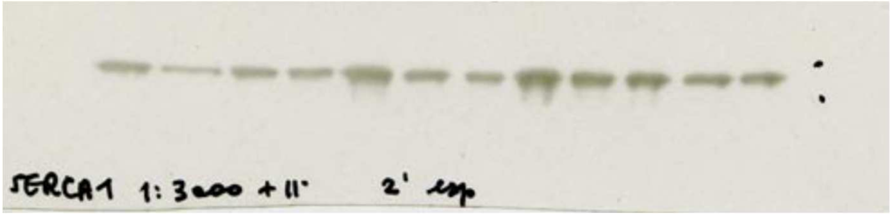

Anti-P-AMPK WB2

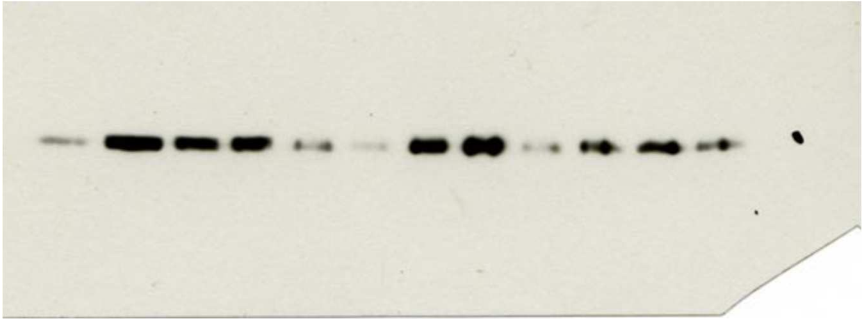

Anti-AMPK WB2

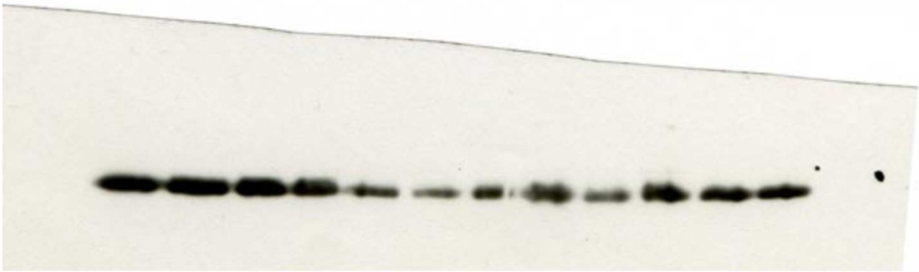

Figure 5: EDL muscle

Red Ponceau WB 1

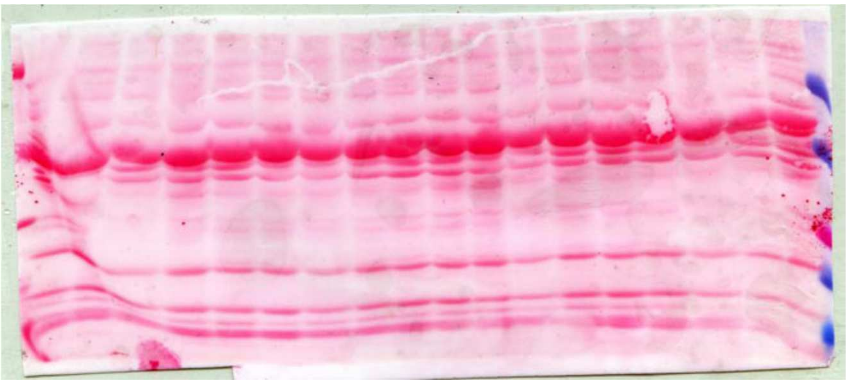

Loading:

1. MW
2. Adult (lane running problem)
3. Adult
4. Adult
5. Old
6. Old
7. Old
8. Old+Cu
9. Old+Cu
10. Old+Cu
11. Old+Cu
12. Old+Cu
13. Old+Cu
14. Old
15. Old
16. Old
17. Adult
18. Adult
19. Adult
20. MW

Anti-Grp94 WB1

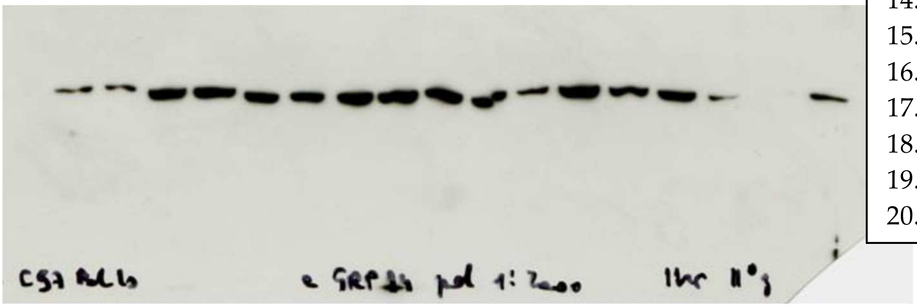

Anti-P-AMPK WB1

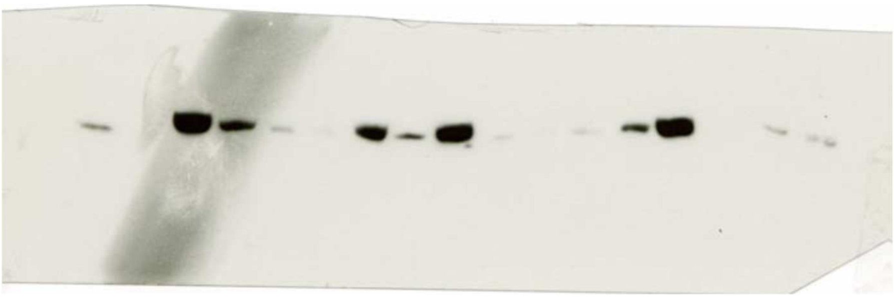

Anti-AMPK WB1

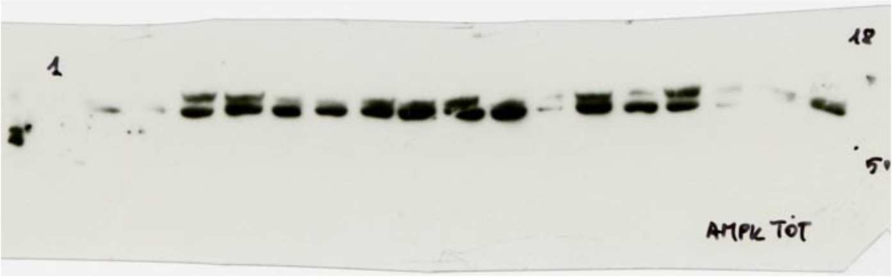

Figure 5 : EDL muscle
Red Ponceau WB2

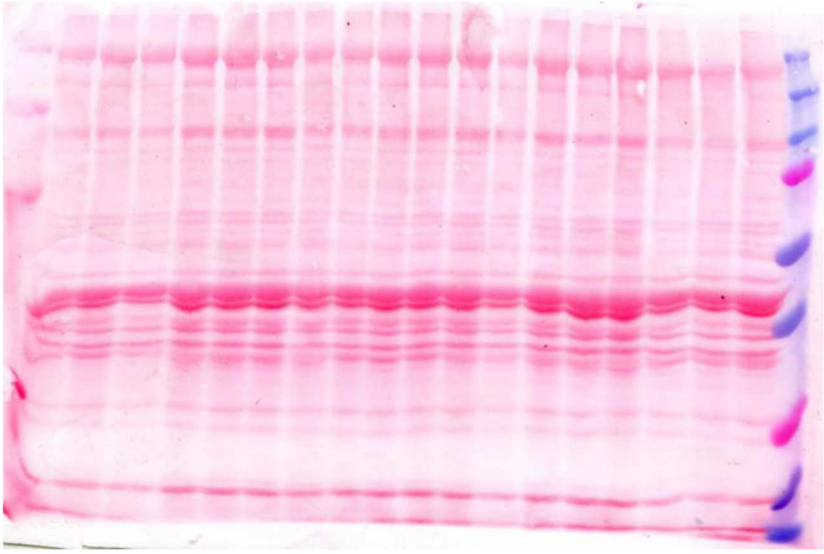

Anti-desmin WB2

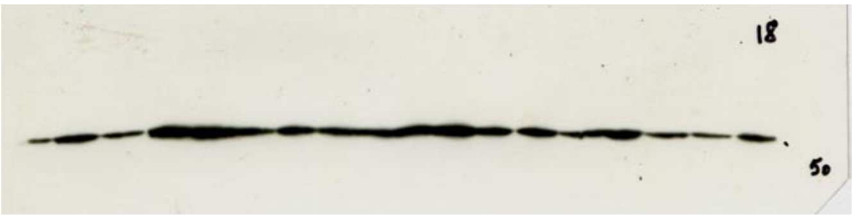

Anti-nNOS WB2

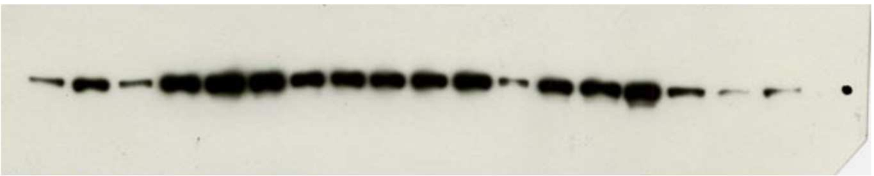

Loading:
1. MW
2. Adult
3. Adult
4. Adult
5. Old
6.Old
7.Old
8. Old+Cu
9. Old+Cu
10. Old+Cu
11. Old+Cu
12. Old+Cu
13. Old+Cu
14. Old
15. Old
16. Old
17. Adult
18. Adult
19. Adult
20. MW

Anti-SERCA1 WB2

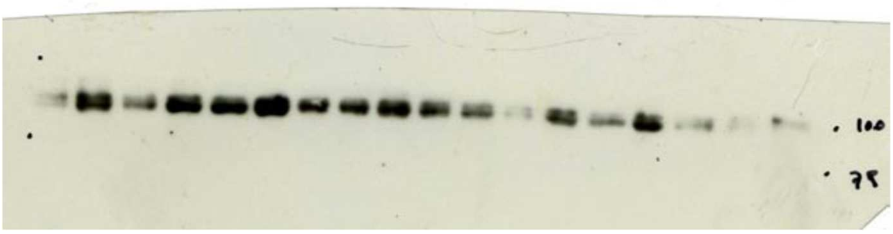

Figure 5 : EDL muscle
Red Ponceau WB3

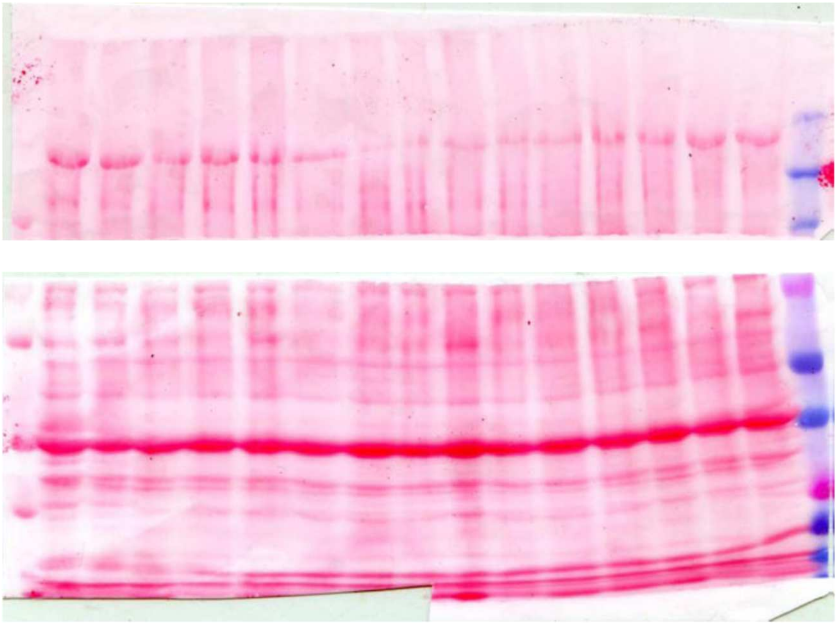

Loading:
1. MW
2. Adult
3. Adult
4. Old
5.Old
6.Old
7. Old+Cu
8. Old+Cu
9. Old+Cu
10. Old+Cu
11. Old+Cu
12.
13.
14.
15.
16.
17. MW

Anti-dystrophin WB3

First exposure

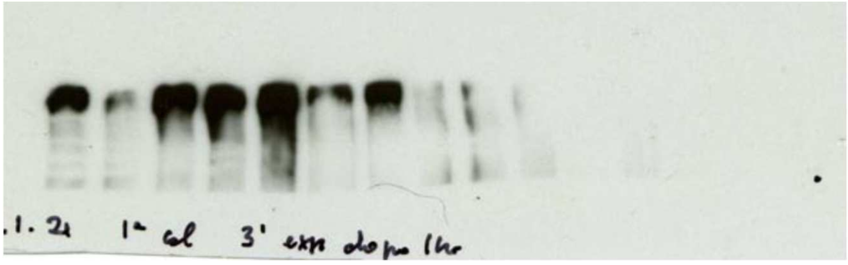

Second exposure

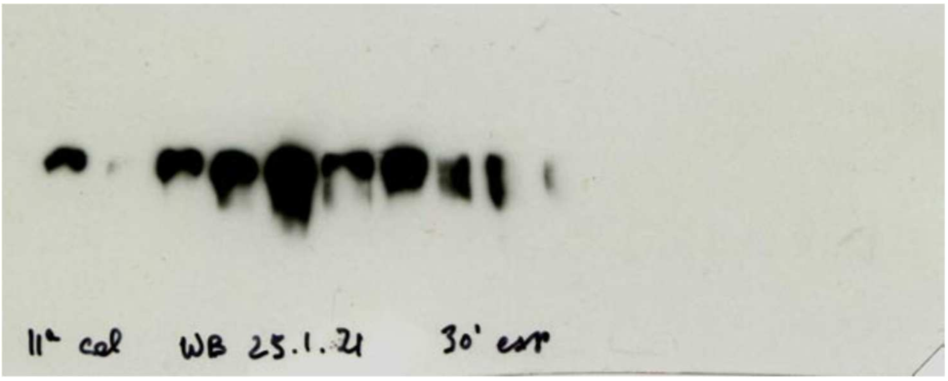

Figure 5: EDL muscle

Red Ponceau WB4

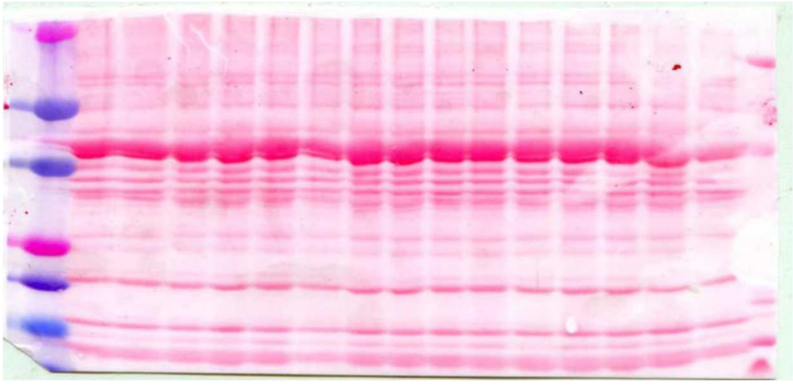

Anti-melusin WB4

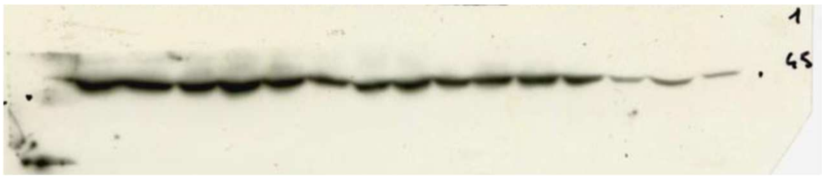

Loading:
1. MW
2. Adult
3. Adult
4. Old
5. Old
6.Old
7. Old+Cu
8. Old+Cu
9. Old+Cu
10. Old+Cu
11. Old+Cu
12. Old
13. Old
14.
15.
16.
17. MW

Figure S7 Panel A (left and middle blots)

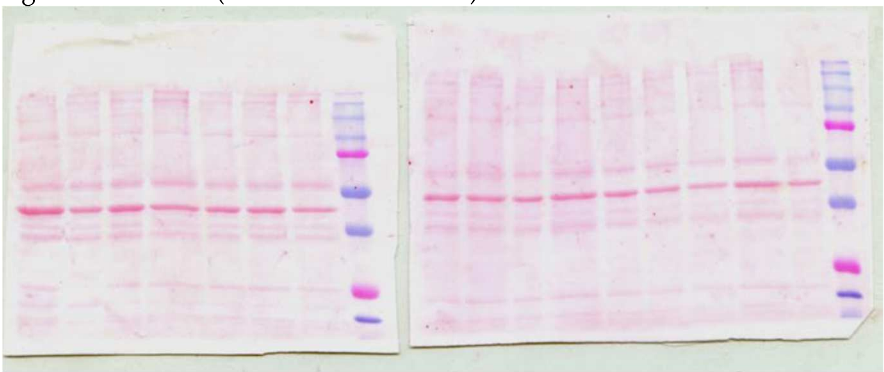

Anti-Tropomyosin

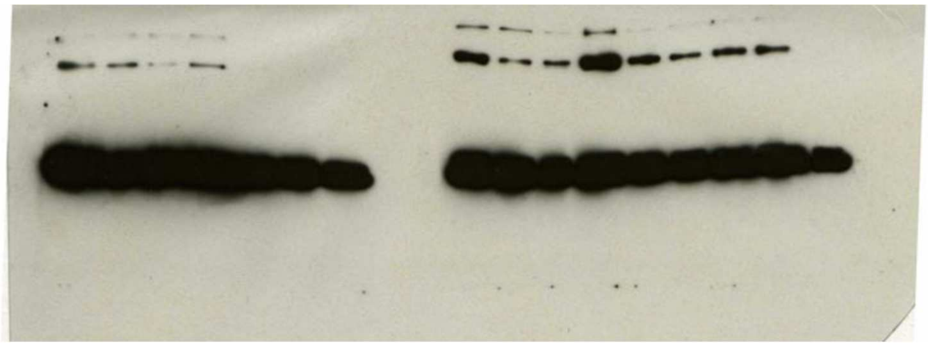

Panel A (right blot)

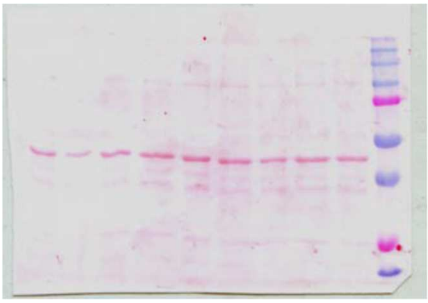

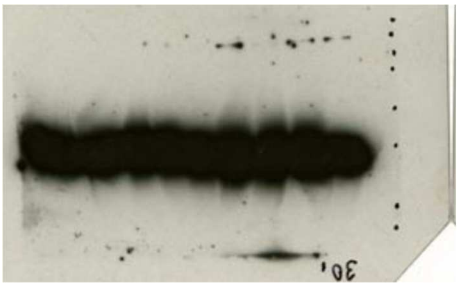

Loading:

Left gel

1. Adult 10ScSn soleus
2. Adult 10ScSn soleus
3. Adult 10ScSn soleus
4. Old 10ScSn soleus
5. Old 10ScSn soleus
6.
7.
8. MW

Right gel

1. Adult mdx diaphragm
2. Adult 10ScSn soleus
3. Adult 10ScSn soleus
4.
5.
6.
7.
8.
9.
10.

Bottom gel

1.
2.
3.
4. Old 10ScSn soleus
5. Old 10ScSn soleus
6. Old 10ScSn soleus
7. Old+Cu 10ScSn soleus
8. Old+Cu 10ScSn soleus
9. Old+Cu 10ScSn soleus
10. MW

Figure 7S Panel B already shows intact original blots.
